# Emerging Technologies and Synergies for Airborne and Space-Based Measurements of Water Vapor Profiles

**DOI:** 10.1007/s10712-017-9448-9

**Published:** 2017-11-21

**Authors:** Amin R. Nehrir, Christoph Kiemle, Mathew D. Lebsock, Gottfried Kirchengast, Stefan A. Buehler, Ulrich Löhnert, Cong-Liang Liu, Peter C. Hargrave, Maria Barrera-Verdejo, David M. Winker

**Affiliations:** 1 NASA Langley Research Center, Hampton, VA 23681 USA; 2 DLR, Institut für Physik der Atmosphäre, 82234 Oberpfaffenhofen, Germany; 3 Jet Propulsion Laboratory, California Institute of Technology, Pasadena, CA 91109 USA; 4 Wegener Center for Climate and Global Change (WEGC) and Institute for Geophysics, Astrophysics, and Meteorology/Inst. of Physics, University of Graz, Graz, 8010 Austria; 5 Center for Earth System Research and Sustainability (CEN), Meteorological Institute, Universität Hamburg, 20146 Hamburg, Germany; 6 Institute for Geophysics and Meteorology, University of Cologne, Pohligstr. 3, 50969 Cologne, Germany; 7 National Space Science Center (NSSC), Chinese Academy of Sciences, 100190 Beijing, China; 8 School of Physics & Astronomy, Cardiff University, 5 The Parade, Cardiff, CF24 3AA UK; 9 Forschungszentrum Jülich, Wilhelm-Johnen-Straße, 52428 Jülich, Germany

**Keywords:** Remote sensing, Water vapor profiles, Atmospheric science, Lidar, Differential absorption lidar, Radar, Differential absorption radar, Microwave occultation, Hyperspectral microwave, Emerging technology

## Abstract

A deeper understanding of how clouds will respond to a warming climate is one of the outstanding challenges in climate science. Uncertainties in the response of clouds, and particularly shallow clouds, have been identified as the dominant source of the discrepancy in model estimates of equilibrium climate sensitivity. As the community gains a deeper understanding of the many processes involved, there is a growing appreciation of the critical role played by fluctuations in water vapor and the coupling of water vapor and atmospheric circulations. Reduction of uncertainties in cloud-climate feedbacks and convection initiation as well as improved understanding of processes governing these effects will result from profiling of water vapor in the lower troposphere with improved accuracy and vertical resolution compared to existing airborne and space-based measurements. This paper highlights new technologies and improved measurement approaches for measuring lower tropospheric water vapor and their expected added value to current observations. Those include differential absorption lidar and radar, microwave occultation between low-Earth orbiters, and hyperspectral microwave remote sensing. Each methodology is briefly explained, and measurement capabilities as well as the current technological readiness for aircraft and satellite implementation are specified. Potential synergies between the technologies are discussed, actual examples hereof are given, and future perspectives are explored. Based on technical maturity and the foreseen near-mid-term development path of the various discussed measurement approaches, we find that improved measurements of water vapor throughout the troposphere would greatly benefit from the combination of differential absorption lidar focusing on the lower troposphere with passive remote sensors constraining the upper-tropospheric humidity.

## Introduction

The dominant source of uncertainty in climate sensitivity estimates has been traced to model diversity in the response of clouds to climate change (Bony and Dufresne 2005; Vial et al. 2013). Narrowing this uncertainty and improving the confidence of climate projections represents one of the greatest challenges faced by the Earth science community, suggesting better constraints on the magnitude of cloud feedbacks are key. While we have had little success in reducing the range of estimated climate sensitivity, we have greatly improved our knowledge of the processes involved. Fundamental mechanisms behind longwave feedbacks due to deep tropical clouds are better understood (Hartmann and Larson 2002), although questions remain about the importance of mesoscale organizing processes (Wing et al. 2017; Wing and Emanuel 2014; Mauritsen and Stevens 2015; Mapes et al. 2017). Shallow clouds are mediated more by atmospheric circulation than by microphysical processes. Petters et al. (2013) performed large-eddy simulation (LES) studies on the sensitivity of overcast stratocumulus liquid water path (LWP) to the humidity of the air in the free troposphere just above cloud top, finding that radiative and evaporative cooling at the cloud top are highly sensitive to the moistness of the free troposphere above cloud top. Realistic variations in moisture were found to have effects on LWP similar to or larger than those expected from perturbations in cloud condensation nuclei. This parallels the known importance of humidity for the development of deeper convection (James and Markowski 2010).

Modeling across scales suggests that many aspects of circulation—the depth of the marine boundary layer (BL), the moisture gradient between the sea surface and the free troposphere, the rate of subsidence, etc.—will change in response to a warming climate. As the community gains a deeper understanding of the processes involved in regulating shallow clouds and their radiative effects, there is a growing appreciation of the critical role played by small fluctuations in water vapor in and above the marine boundary layer and the coupling of water vapor and atmospheric circulations.

In the tropics, water vapor has a maximum variability in the lower troposphere near 800 hPa (Holloway and Neelin 2009). Recent observations (Nuijens et al. 2009, 2017) and LES studies (Seifert et al. 2015) of trade cumulus regimes suggest that subtle variations in humidity in the lower troposphere play a dominant role in convective development and in buffering aerosol-cloud radiation interactions. Mixing processes acting to redistribute water vapor between the free troposphere and the marine boundary layer can impact the albedo and frequency of shallow marine clouds. Lower tropospheric mixing has been seen to scale strongly with feedbacks in models (Sherwood et al. 2014) but is poorly constrained by observations. Proxies for small- and large-scale mixing are given by the structure and depth of the moist layer over the tropical oceans. Better constraints on water vapor anomalies in the lower troposphere-requiring higher vertical (~ ≤ 0.3 km) and horizontal (~ ≤ 10 km) resolution and accuracy (≤ 2%)—would improve understanding in all of these areas and constitute a tremendous advance (ISSI Workshop on Shallow clouds and water vapor, circulation and climate sensitivity).

In summary, progress on the role of atmospheric water and circulation requires improved understanding of underlying processes by better observations of water vapor, clouds, and winds, especially in the lower troposphere. A high vertical measurement resolution is needed because strong vertical moisture gradients at the top of the mixed layer and in the free troposphere aloft can strongly influence radiation and the development of both shallow and deep convection (Stevens et al. 2017). Generally, water vapor measurements are challenging, both because water vapor is highly variable in space and time on a large range of scales, and because the physics of the various measurement approaches used to date fundamentally limits each instrument’s performance in different ways. The ideal hygrometer still does not exist, but interesting new technologies are emerging that show promise in overcoming limitations of current measurement approaches. It is therefore timely to explore their capabilities and synergies, and to discuss their advances with respect to the conventional measurement techniques.

In situ hygrometers, mainly deployed on land stations, buoys, ships, balloons or aircraft can be highly accurate but suffer from poor representativeness as they offer point or line measurements in space or time. They are very irregularly distributed around the globe: Their density is poor over remote regions and oceans, and vertical profiles are rare. Spectrometers on satellites provide global coverage yet are impeded by low measurement sensitivity in the lower troposphere due to blurring by the water vapor column aloft. Their complex retrieval is sensitive toward thin clouds or aerosol layers such that measurement biases may arise if clouds and aerosol remain undetected, or if they are accounted for at incorrect altitudes. Their data have coarse spatial resolution, particularly in the vertical, limb sounding being no option in the lower troposphere. Better spatial resolution, combined with the interesting possibility to trade-off vertical versus horizontal resolution versus measurement precision, in order to optimize the measurements to the needs of their users/communities, can be provided by active remote sensing such as differential absorption lidar (DIAL) and (in the future) radar (DAR). DIAL and DAR come at the cost of higher instrument complexity, and a certain loss of coverage since most active instruments have no scanning capability in order to keep complexity within reasonable limits. Clouds are obstacles for DIAL, yet the lidar sensors’ field of view is sufficiently small that every cloud gap provides an opportunity for vertical profiling. An upcoming cost-efficient technique is radio/microwave (signal) occultation. Since the measurement geometry is similar to limb sounding, however, the benefit for profiling humidity in the planetary boundary layer is limited due to the inherent broad horizontal averaging kernels. The value of this new technique will grow with the number of exploitable occultation events. Lastly, new detector technologies open the possibility for hyperspectral microwave sensors that can support thousands of spectral channels with little to no sensitivity to cloud contamination. This paper highlights these new or improved technologies for measuring lower tropospheric water vapor presented at the ISSI Workshop on Shallow clouds and water vapor, circulation and climate sensitivity, and their expected added value to current observations. The potential synergies and added benefits of each measurement technique compared to existing observational methods are also discussed.

## Differential Absorption Lidar

The differential absorption lidar (DIAL) technique was first used to measure lower tropospheric water vapor by Schotland (1966), just a few years after the discovery of the laser, and it has since been used to measure a number of trace gases in the atmosphere. So the technique is not new, yet currently benefits from significant technological advances. It utilizes atmospheric molecular (Rayleigh) and aerosol (Mie) backscattered return signals from a tunable single-frequency, pulsed laser to directly measure range resolved profiles of water vapor molecular number density N(R). Two spectrally close laser pulses (typical 10–100 ns pulse width) are transmitted to the atmosphere near simultaneously (0.2–1 ms separation) with one pulse tuned to the center or wing of a gas absorption line, called the online (λ_on_), and the second pulse, called the off-line (λ_off_), tuned to a less absorbing spectral location. The separation between the online and off-line wavelengths is typically < 1 nm to minimize biases resulting from differences in atmospheric backscatter and extinction coefficients. The retrieval of concentration profiles is achieved using the DIAL equation,

$$ N\left( R \right) = \frac{1}{{2\Delta R\left( {\sigma_{\text{on}} - \sigma_{\text{off}} } \right)}}\ln \left[ {\frac{{P\left( {\lambda_{\text{on}} ,R} \right) P\left( {\lambda_{\text{off}} ,R +\Delta R} \right)}}{{P\left( {\lambda_{\text{off}} ,R} \right) P\left( {\lambda_{\text{on}} ,R +\Delta R} \right)}}} \right], $$

which exploits the differential attenuation of the backscattered return signals (*P*_*λ*on_, *P*_*λ*off_ (*W*)) between the online and off-line wavelengths along with a priori knowledge of the differential absorption cross section Δ*σ* (cm^2^) over a range bin Δ*R* (m) (Schotland 1974).

Given the laser on–off pulse pair temporal and spectral separation are sufficiently small (≤ 1 ms and < 1 nm, respectively), it can be assumed that the atmospheric volume scattering and extinction coefficients are constant between the online and off-line atmospheric return signals. As a result, DIAL from aircraft and space have the potential for high accuracy measurements of trace gases, in particular, water vapor throughout the troposphere, as they are self-calibrating and not prone to bias resulting from aerosol and cloud contamination.

### Measurement Capabilities

**Fig. 1:**
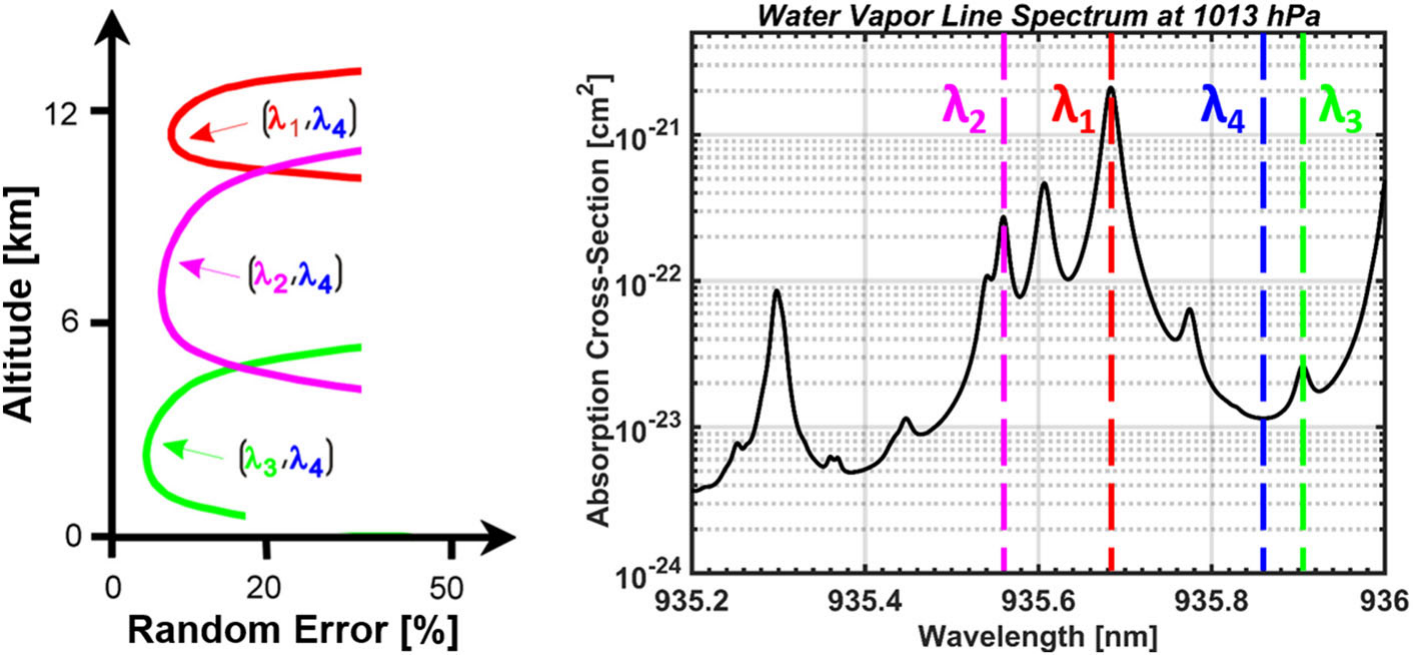
A space-based DIAL transmitter will require transmission of two or more online wavelengths to adequately measure the large water vapor dynamic range from the upper troposphere down to the near-surface atmosphere. The different online wavelengths experience different water vapor optical thicknesses, resulting in different measurement sensitivities. For WALES and HALO (see Sect. 2.2), three combinations of signals using three wavelength pairs result in a composite water vapor profile throughout the troposphere, with expected relative random error profiles depicted in the left panel (ESA 2001). Hard target returns from the surface can be used to measure the full column at each wavelength using the integrated path DIAL approach (IPDA). Water vapor measurements from the lowest retrievable range bin can be extended down to the surface using IPDA. Measurements/missions focused on the mid-lower troposphere would benefit from the reduced number of required online wavelengths as well as from the added flexibility in the transmitter design by having additional access to weaker absorption lines in the 820 and 720 nm spectral bands

Water vapor DIAL has similar performance over land and ocean as it is insensitive to surface emissivity. Further, water vapor profiles can be retrieved during day and night, at all latitudes, and during all seasons. Multiple online wavelengths can be implemented to increase the dynamic range required for measurements in different climates as well as for full tropospheric profile measurements from the upper troposphere and lower stratosphere (UTLS) down to the tropical marine layer (Fig. 1). Generally, Lidar on aircraft can efficiently probe target regions of interest for the study of particular atmospheric processes in two or three (when using a scanner) spatial dimensions. When installed nadir-viewing, another advantage arises from the fact that air density, and partly also humidity and aerosol concentration, increases exponentially with range below the aircraft, thus partly compensating the decrease in backscatter signal intensity that is proportional to the range-squared. Airborne water vapor DIAL systems have been developed over the past 20 years with focus on tropospheric and UTLS measurements from local to synoptic scales for weather and climate studies (Higdon et al. 1994; Bruneau et al. 2001; Browell et al. 1997; Ehret et al. 1998, 1999; Poberaj et al. 2002; Wirth et al. 2009; Ferrare et al. 2004). Lidar technology developments have evolved at DLR, Germany, to enable advanced DIAL systems which operate in the strongly absorbing 935-nm water vapor band. These systems have been developed as airborne demonstrators for future satellite missions and have been deployed within many large-scale field experiments. They have demonstrated measurement capability in the UTLS region, as well as in cirrus clouds (Kiemle et al. 2008; Groß et al. 2014). Recently, airborne water vapor lidar observations focused on tropical shallow convective environments have been reported (Kiemle et al. 2017), while earlier work has demonstrated the value of airborne lidar to characterize the variability of humidity and of latent heat fluxes within the convective boundary layer over land (Kiemle et al. 2011).

**Fig. 2:**
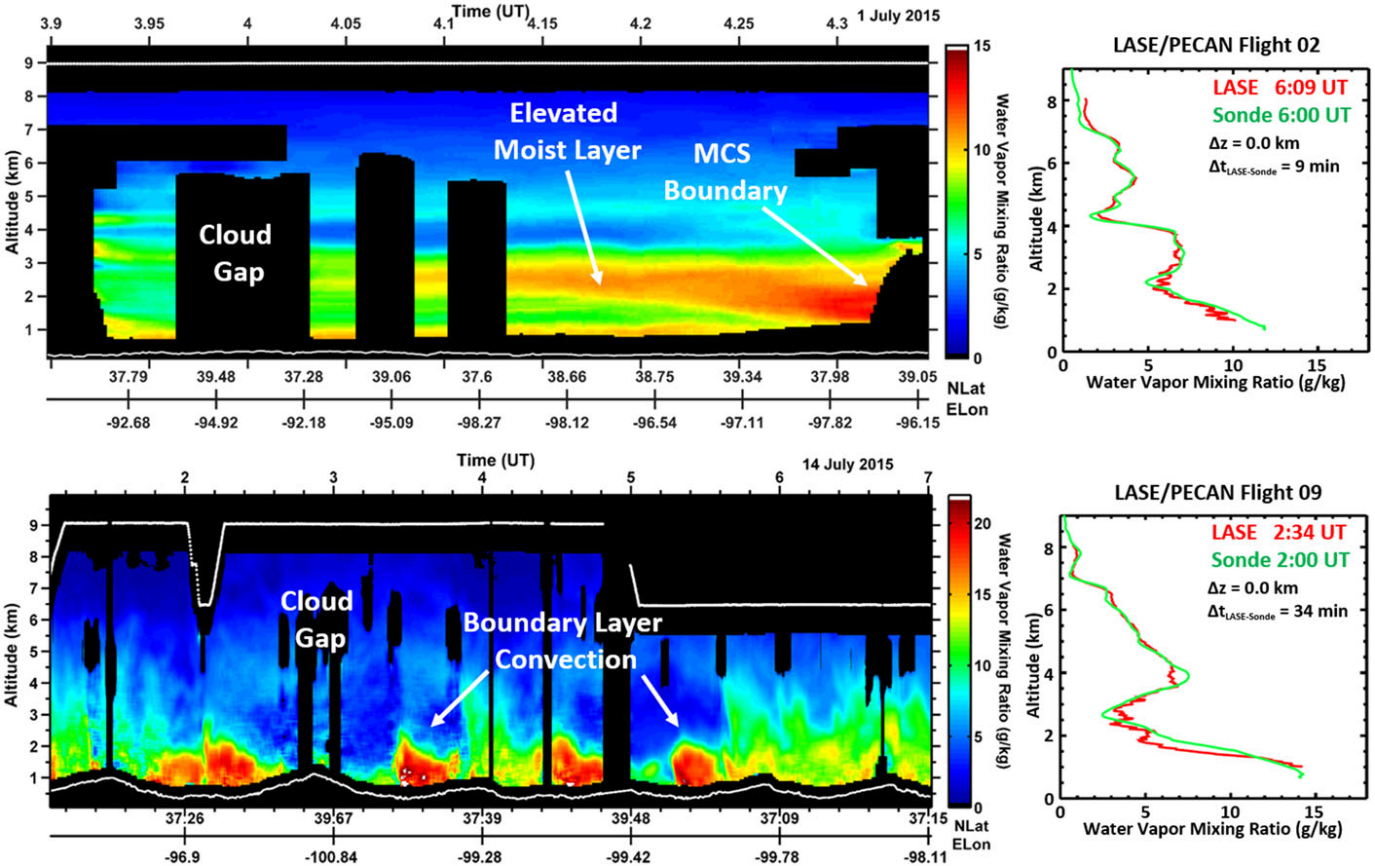
Examples of water vapor profiles measured by LASE in advance of two separate mesoscale convective systems (MCS) during the 2015 PECAN mission over the US central Great Plains. The LASE time series data (left panels) reveal strong spatial variability ahead of high impact weather systems that are not resolved by passive systems. Overpasses of PECAN ground sites by the NASA DC-8 aircraft show good agreement between LASE and coincident radiosonde measurements (right panels). The sonde comparison shown for PECAN flight 02 occured outside of the corresponding time series chart to the left. The white lines at the top and bottom of the time series charts represent the aircraft flight path and surface height, respectively. The blacked out sections in the time series charts lack data due to lidar beam extinction by cloud. Δz and Δt represent the spatial and temporal separation between the sond launch and aircraft overpass time and location, respectively

Developed at NASA as an initial step toward a space-based water vapor DIAL, the airborne Lidar Atmospheric Sensing Experiment (LASE) has also demonstrated that profile measurements of water vapor can be made to an accuracy of better than 6% or 0.01 g/kg (whichever is larger) throughout the troposphere with 330 m and 14 km vertical and horizontal resolution, respectively (Browell et al. 1997; Ferrare et al. 2004). Figure 2 shows recent measurements from LASE, which has now been in operation for nearly two decades. These results are over the central Great Plains during the Plains Elevated Convection At Night (PECAN) mission and illustrate the high vertical resolution achievable and high accuracy relative to radiosonde profiles. Use of online/off-line surface reflection signals provides column water vapor and also for extending the water vapor profiles to within 100 meters above the surface, although not implemented in the figures shown here. Water vapor DIAL has the potential to provide high accuracy and high vertical resolution profiles from space, particularly near the surface where retrievals from passive and active microwave sensors struggle the most. Future prospects for Earth observing systems focused on atmospheric dynamics will greatly benefit from complementary measurements between active DIAL sensors which have greatest sensitivity to the near the surface atmosphere, and passive/active microwave and occultation approaches (discussed below) whose averaging kernels are weighted to the mid-upper troposphere. The combination of active and passive retrievals has the potential to result in high-resolution water vapor profiles from the surface to the stratosphere.

**Fig. 3:**
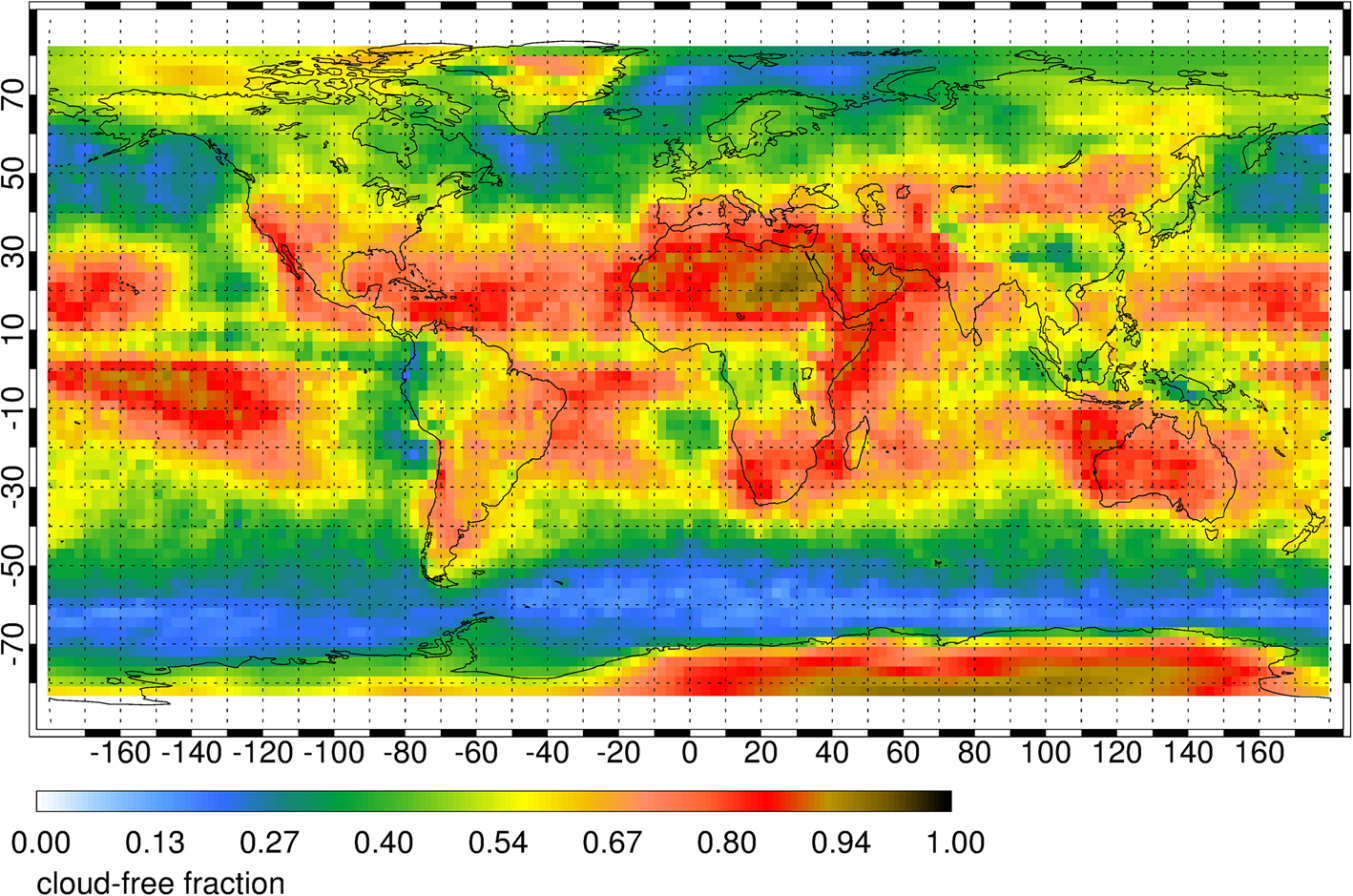
Global map of annual mean CALIPSO-derived cloud-free fraction. A CALIPSO column is defined to be cloud-free whenever its total measured cloud optical depth is less than 1. The cloud statistics were compiled from data collected in 2007. In 2007, the annual global cloud-free fraction was 53.5%, suggesting that the retrieved water vapor profiles of a space-based DIAL could extend down to the surface for over half of the total retrieved profiles and thereby substantially add value to the next generation of atmospheric remote sensors targeting tropospheric dynamics

Lidar is characterized by small footprints relative to radars and radiometers (on the order of 100 m) and can profile above low clouds and below optically thin cirrus. Cloud masking and shot averaging allows probing between broken boundary layer clouds. Opaque clouds are the primary limiting factor to the number of useful observations retrieved from space-based active and passive remote sensors. Similar to the methods carried out by Kiemle et al. (2015), the number of useful water vapor DIAL profile measurements that extend down to the surface can be determined using the cloud fraction measurements from the Cloud-Aerosol Lidar and Infrared Pathfinder Satellite Observations (CALIPSO) mission. CALIPSO level-2 version 3-01 cloud layer products are aggregated into a 278 × 278 km^2^ (2.5° × 2.5°) grid where a cloud optical depth (OD) threshold of 1 (86.5% two-way attenuation) is applied to the grid as a means to differentiate between clear and cloudy (cloud OD > 1) conditions. Figure 3 shows the global map of CALIPSO-derived cloud-free fraction averaged over 12 consecutive months in 2007. The resulting cloud-free fraction map shows most favorable conditions along the northern and southern subtropics, a geographic area where water vapor has a maximum variability in the lower troposphere (Holloway and Neelin 2009) and subtle variations in lower tropospheric humidity play a dominant role in convective development and in buffering aerosol–cloud–radiation interactions. DIAL measurements are impacted by optically thin clouds with OD < 1; however, the signals are further attenuated, not fully obstructed. In consequence, more horizontal and/or vertical averaging can provide profiles with accuracy and precision similar to cloud-free profiles. The coarse cloud-mask blurs many of the small cloud-free regions that appear in higher-resolution space lidar analyses (see, e.g., Kiemle et al. 2015, Fig. 3 for CALIPSO cloud stats). The likely reduction in average OD using this blurred cloud mask is not quantified here, however, the effect is less detrimental to instruments with small field of view such as lidar by enabling profiling within small cloud gaps, and is expected to be most pronounced in regions with (deep) convective clouds and fair weather cumulus that have sharp edges, particularly in the tropics.

Simulations based on the experience gained with airborne demonstrators at NASA and DLR have demonstrated the potential for high-resolution water vapor DIAL profiling from a LEO satellite throughout the troposphere (Ismail and Browell 1989; Di Girolamo et al. 2008). The Water Vapour Lidar Experiment in Space (WALES) is a European Space Agency Earth Explorer mission proposed in the early 2000s targeting profile measurements of tropospheric water vapor using the DIAL technique (ESA 2001). Unfortunately, WALES was not selected for further development due to cost and technical risk associated with the laser under consideration; however, several feasibility studies were carried out and demonstrated the utility of such measurements for weather and climate applications. A concept study by Gerard et al. (2004) assessed the performance of a space-based water vapor DIAL relative to passive infrared measurements such as from IASI (Infrared Atmospheric Sounding Interferometer). The results of the study indicated that for a fixed error threshold, the WALES water vapor DIAL concept under study would extend water vapor measurements to higher altitudes than for IASI, with improved vertical resolution near the surface (Gerard et al. 2004). Di Girolamo et al. (2008) also carried out an end-to-end simulation of a WALES like lidar to assess the systematic and random error performance under a wide range of atmospheric conditions. The results confirmed the capability of a space-based water vapor DIAL of accurately measuring the water vapor and aerosol structure from the UTLS down to the surface with better than ± 5% bias and ± 20% random error. The measurement requirements resulting from the space-borne WALES mission study (Gerard et al. 2004; DiGirolamo et al. 2008) are shown in Table 1. Although the concept study was carried out over a decade ago, the general sampling and accuracy requirements for a space-based DIAL for the application of improving Numerical Weather Prediction is still valid. The third column represents target measurement requirements (resulting from the 2016 Shallow clouds and water vapor, circulation and climate sensitivity Workshop) needed to advance our understanding of the interplay between shallow clouds, atmospheric circulation, and climate sensitivity. Although airborne DIALs have demonstrated the high spatial resolutions called out for cloud/climate studies, the improved vertical and spatial sampling requirements pose a challenge for space-based observations with current technologies.

**Table 1:**
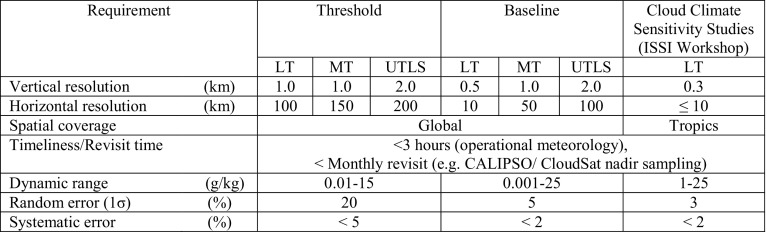
Space-borne water vapor DIAL observational requirements adapted from the WALES lidar mission concept (Gerard et al. 2004)

Measurement requirements for cloud and climate sensitivity studies result from the 2016 Shallow clouds and water vapor, circulation and climate sensitivity workshop (Stevens et al. 2017; Bony et al. this issue). Dynamic range requirements for DIAL missions targeting lower tropospheric measurements can be relaxed to cover those ranges typically observed from the mid-troposphere (< 10 km) down to the surface. Narrowing the focus of a mission to measurements in the mid-lower troposphere opens the possibilities of new laser technologies with reduced complexity and hence risk. Lower troposphere (LT) ranges from ~ 0 to 5 km, mid-troposphere (MT) ranges from ~ 5 to 10 km, and upper troposphere/lower stratosphere (UTLS) ranges from ~ 10 to 17 km

**Fig. 4:**
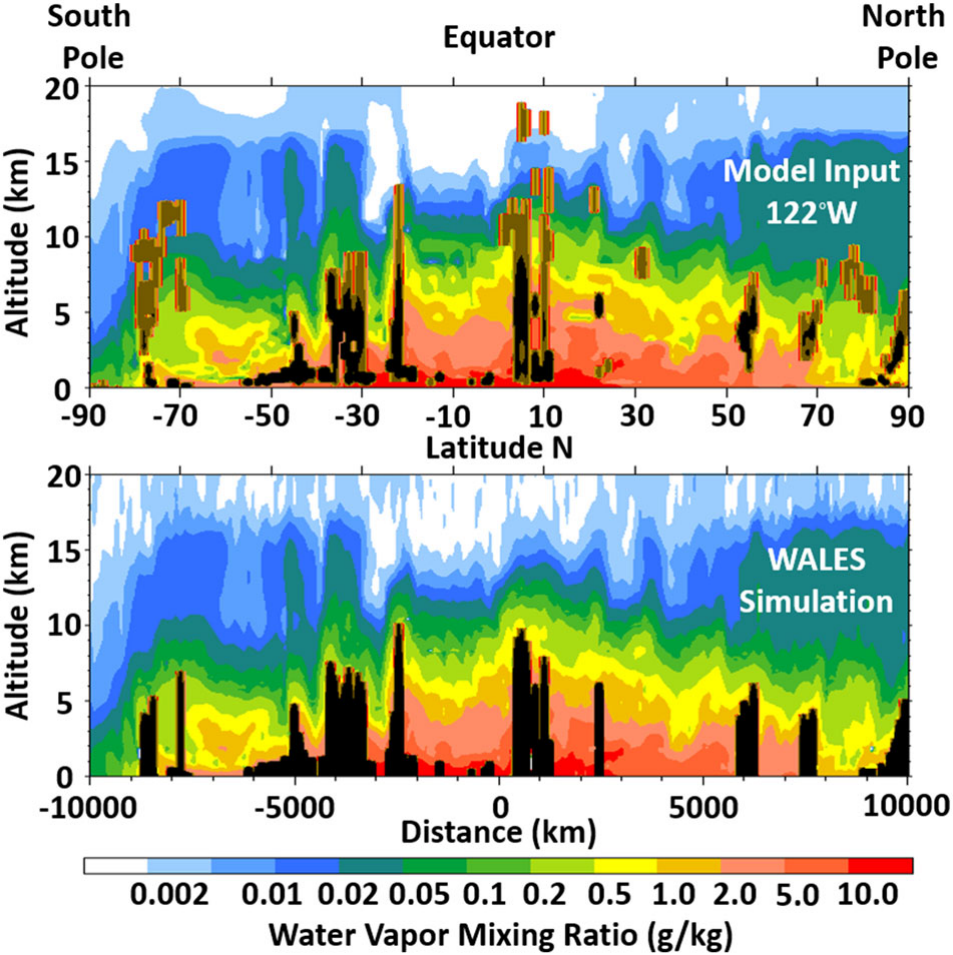
Pole-to-pole water vapor cross section along 122°W (mainly over the Pacific Ocean and Canada) representing the “true” atmospheric state for input into the WALES simulation model (top), and output of this model, i.e., what this space lidar would be able to observe (bottom). Information below thick clouds is lost (black), yet the tropospheric water vapor distribution and variability is very well reproduced. In total, 83% of the simulated cloud-free observations below 15 km agree to within 20% with the model reference

The high accuracy and precision capability of a space-based water vapor DIAL, WALES, is further demonstrated in a model-simulation comparison shown in Fig. 4. A single global latitude slice from a Numerical Weather Prediction (NWP) model run at 122 W longitude on May 24, 2001, is used to define the water vapor field that is ingested into the WALES lidar simulator. Thin and thick clouds in the NWP model are depicted by the brown and black feature masks, respectively. These feasibility studies, including the model-simulation comparison in Fig. 4, demonstrate the ability of the DIAL technique to measure fine-scale water vapor features from the upper troposphere down to the near-surface atmosphere with high precision and low bias in clear and cloudy skies during both day and night conditions. These capabilities have been further realized via airborne demonstrators (airborne WALES and LASE) that match and often exceed the WALES space mission requirements (as well as those required for shallow cloud studies) on precision and accuracy, although over much shorter horizontal averaging scales. The requirements for space-based observations of the tropical marine boundary layer (with the resolution required to improve our understanding of cloud-climate feedbacks) are challenging and would require substantial technological investments in the near term. However, airborne DIAL measurements with capabilities that surpass the aforementioned measurement requirements can be used in the interim to conduct process oriented field studies that supplement space-borne observations that lack the required spatial resolution/sampling, but offer global coverage over long time series.

### Technology Readiness

NASA and DLR have fielded airborne water vapor DIAL systems for targeted process studies over the last two decades and have successfully demonstrated key elements required for accurately measuring water vapor profiles from airborne platforms. While laser energy and spectral purity requirements for airborne and space-based DIAL measurements are fulfilled by LASE and WALES, the electrical efficiencies for both systems are unsuitable for scaling to space. Advances in laser and lidar technologies leveraged from previous successful missions such as CALIPSO as well as ongoing developments such as EarthCare, ADM-Aeoulus, ICESAT-II, MERLIN, and CALIPSO follow-on mission concepts (Albert et al. 2015) have enabled a new class of airborne water vapor DIALs that serve as prototypes for future satellite missions. NASA is currently developing a new airborne DIAL to fly on a variety of mid-to-high altitude platforms for profile measurements of water vapor and aerosols and clouds from the surface to the lower stratosphere. This development will serve as a prototype for future space-based water vapor DIAL missions. Recent investments in airborne DIAL have led to reduction in size, weight, and power budgets commensurate with those required for future space-based water vapor DIAL missions. Further technology investments are required, however, in the areas of high-power lasers, optical receivers, and detectors to scale technology used in current airborne systems to be suitable for satellite DIAL missions with reasonable cost and reduced risks of failure.

The LASE and WALES DIAL systems operate at 820 and 935 nm, respectively. The 935 nm spectral band was chosen for WALES to allow for full tropospheric profile measurements from the UTLS down to the surface (Wirth et al. 2009). The 820 nm spectral band was chosen for the LASE system to increase sensitivity to the near-surface atmosphere and utilize mature Ti/Sapphire laser technology (Moore et al. 1997; Browell et al. 1997, 1998). Furthermore, this spectral band was the only region accessible by means of direct laser generation at the time, opposed to WALES which was developed in the mid-2000s and utilizes parametric conversion to generate coherent laser light in the 935 nm spectral band. The newest DIAL system under development at NASA is the High Altitude Lidar Observatory (HALO), planned for aircraft deployment on the NASA King Air platform in 2018. HALO is configured to fly on the NASA ER-2 aircraft, which will provide a viewing geometry able to simulate the performance of an eventual satellite instrument, but will also be capable of flying on smaller aircraft for targeted process studies. HALO is a multi-function lidar and is designed as a technology test bed for future space-based trace gas DIAL missions. To that end, HALO will have the capability to operate at the 935, 820, and 720 nm water vapor bands to allow for risk reduction of key water vapor DIAL technologies (namely the optical receiver filters, detectors, and transmitter technologies) required for future satellite missions. HALO is much more compact and electrically efficient compared to its predecessor LASE system and employs a more suitable set of technologies for scaling to space.

WALES operates at 935 nm, providing coverage throughout the troposphere. For a mission focused on the lower troposphere (below 8–10 km), the 820 or 720 nm spectral bands have advantages. Increased Rayleigh scattering at these shorter wavelengths increases the lidar signal and allows for use of more sensitive detectors with lower noise characteristics. Furthermore, direct generation of laser radiation at these wavelengths based on new laser materials is emerging and would significantly reduce the complexity and improve the efficiency of a laser transmitter required for space-based water vapor DIAL. A mission focused on lower tropospheric measurements would also reduce the number of required online wavelengths, significantly reducing the complexity and cost of a space-based DIAL transmitter, while at the same time increasing the signal to noise ratio due to an effective increase in DIAL wavelength-pair pulse repetition rate.

Targeted investments are needed in the area of single-frequency lasers to increase the technology readiness level (TRL) for space. The WALES airborne demonstrator has achieved the laser pulse energy and spectral purity required for space-based water vapor DIAL measurements; however, the size, weight, and power (SWAP) figures are not scalable to a space-based implementation. HALO laser pulse energies, while sufficient for aircraft applications need to be increased by a factor of 10 to be suitable for space, while maintaining reasonably high electrical wall plug efficiency (> 5–8%). Leveraging emerging technologies in single photon counting detectors could, however, reduce the requirement on laser pulse energy to those currently achieved with the HALO 935 nm laser, which exhibits the SWAP required for a space-based implementation.

Transitioning to a low energy and high pulse repetition rate laser architecture, however, requires the use of narrow field of view receivers and extremely narrow-band and frequency agile optical filters. Technology advancements in narrow-band (1–20 pm full width at half maximum) rapidly tunable (frequency agile) etalons are also required. The solar background noise in DIAL systems is proportional to the spectral width of the band-pass filter in the receiver. Online/off-line wavelength pairs used to sample the targeted water vapor absorption line(s) are typically separated by a few 100 pm (Fig. 1). Rather than accommodating both wavelengths within the receiver band-pass filter, narrow-band frequency agile etalons are needed which can be tuned between the online and off-line wavelengths on a shot-by-shot basis. The spectral width of the etalons can then be as low as 1–20 pm, and if successfully implemented has the potential to significantly increase the daytime measurement signal to noise ratio over high albedo scenes, allowing for boundary layer water vapor measurements with better than 5–10% precision using existing and higher TRL laser architectures. The transmitter and receiver subsystem TRLs using technologies developed for WALES, LASE, and HALO (as well as applicable technologies developed under existing space-programs) are at 4–5 and 5–6, respectively. TRL 4 is component/subsystem validation in a laboratory environment, TRL 6 is system/subsystem model or prototyping demonstration in a relevant end-to-end environment. The aforementioned systems and receiver and transmitter technologies could be matured to be space-flight ready within the next 5–7 years by targeted investments.

## Differential Absorption Radar

Differential Absorption Radar (DAR) is the radar analogue of DIAL. The method exploits the difference in gaseous attenuation between two or more channels closely spaced in frequency. This differential attenuation can be related to the gas content of the atmosphere. A key difference between the DAR and DIAL is in the transmitted frequency and related atmospheric scattering properties. DIAL uses clear-sky molecular and aerosol scattering, whereas DAR uses cloud and precipitation scattering targets, thereby enabling sounding capabilities within clouds, highly complementary to DIAL.

In a general sense the DAR technique can be used to sense any species with an absorption feature in the microwave spectrum. For example, prior studies have proposed to observe surface pressure using channels in the 60 GHz oxygen absorption complex scattered off of the Earth surface (Lin and Hu 2005; Millán et al. 2014) and some initial observations have been made with an airborne prototype (Lawrence et al. 2011). Use of the 22 GHz absorption line has been proposed to observe water vapor within precipitation echoes (Meneghini et al. 2005). Additionally, exploiting the differences in absorption by water vapor in the continuum has been proposed to retrieve water vapor (Ellis and Vivekanandan 2010; Tian et al. 2007).

The application of DAR proposed here utilizes two radar channels in the G-Band adjacent to the 183 GHz water vapor absorption line (Millan et al. 2016; Lebsock et al. 2015). At range *r*, the ratio of the radar reflectivity (*Z*) at two close frequencies (*ν*) near the absorption line is approximately proportional to the water vapor path ($u_{{h_{2} o}}$) between the transceiver and the scattering volume,

$$ \frac{{Z\left( {\upsilon_{1} ,r} \right)}}{{Z\left( {\upsilon_{2} ,r} \right)}} \propto u_{{{\text{h}}_{2} {\text{o}}}} . $$

**Fig. 5:**
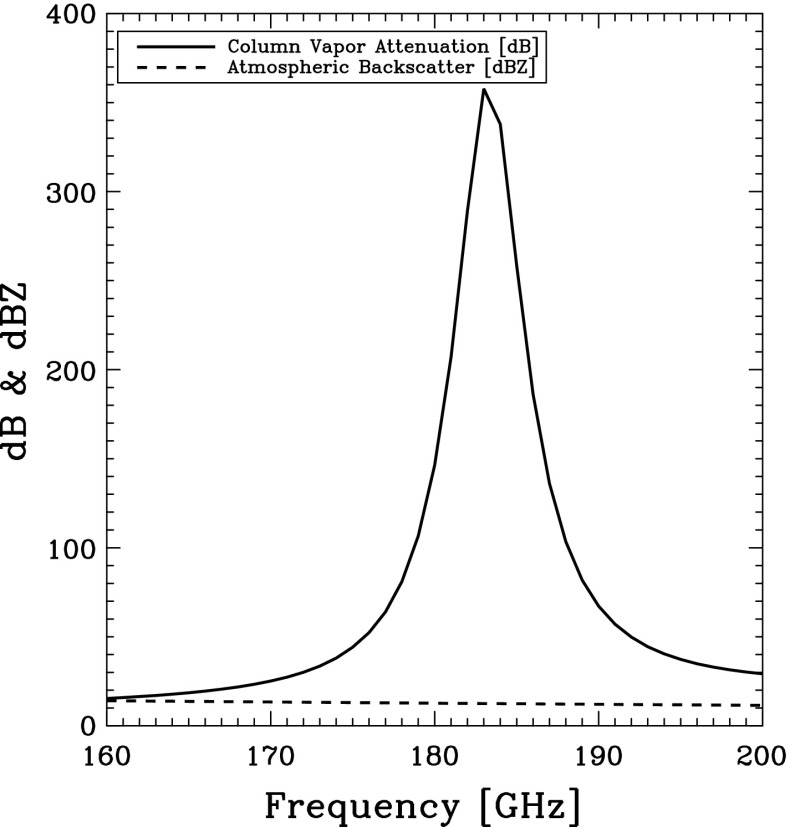
Example of the spectral variation of the column-integrated attenuation due to water vapor and the backscatter due to clouds and precipitation for a trade wind boundary layer. Droplet backscatter variations are essentially negligible relative to water attenuation across the absorption line. Data taken from the simulations of Lebsock et al. (2015)

Here, it has been assumed that variation of extinction properties of the cloud with frequency is much smaller than the variation of the attenuation due to water vapor. In practice, there are small variations in the cloud extinction properties, which can be accounted for. To demonstrate the underlying physics, Fig. 5 shows the spectral variation of the atmospheric attenuation and particle backscatter across the 183 GHz line for a case from the cloud simulations in Lebsock et al. (2015). The column-integrated water vapor attenuation varies by several hundred dB while the column-integrated backscatter varies in a quasi-predictable manner by only 2.5 dBZ.

To provide profiling capabilities a DAR instrument might be implemented as either a range-gated or Frequency Modulated Continuous Wave (FMCW) radar. Water vapor profiles can be obtained by differencing the retrieved partial water vapor path between adjacent range bins and dividing by the range resolution, similar to DIAL retrieval of water vapor concentrations. As a result, the uncertainty in the derived water vapor profiles is an inverse function of the radar range resolution.

In addition to in-cloud profiling capabilities, a downward-looking DAR could provide spatially continuous observations of the column-integrated water vapor (CWV) from the surface reflection, which should be available over all surface types and nearly all atmospheric conditions, with the notable exception of heavy precipitation, which attenuates the signal too strongly.

### Measurement Capabilities

The primary advantage of a water vapor DAR is that it specifically profiles water vapor within clouds. This is a unique measurement capability that cannot be obtained by existing technologies. For example, microwave sounders are sensitive to water vapor in cirrus clouds; however, they have broad weighting functions that encompass both cloud and surrounding clear atmosphere. The observations can further be contaminated by the presence of other clouds. Global Positioning System Radio Occultation (GPS-RO, here below) is also sensitive to water vapor within clouds; however, RO has extremely broad horizontal weighting functions, suffers non-uniqueness near the Earth surface, and has infrequent sampling. Methods that rely on infrared, near-infrared, and visible wavelengths do not penetrate water clouds at all.

Depending on channel selection, a downward-looking water vapor DAR can feasibly sound either high altitude cirrus clouds or low altitude boundary layer clouds. Channels near the absorption line center are required to sound high altitude clouds, whereas channels that attenuate less strongly in the wings of the absorption line are required to sound low altitude clouds. Sampling by a two-channel DAR will be limited by channel selection. The vertical sampling of the CloudSat Cloud Profiling Radar (CPR) may be considered as a best-case sampling capability provided by a DAR within the near future, including the capability to sample Earth’s ubiquitous multi-layered cloud systems. It must be noted that a DAR matching the vertical sampling capability of CloudSat would require a high-power multi-frequency system. More likely, the first DAR will be targeted to a particular altitude range through channel selection.

Initial studies have documented expected uncertainty of a space-borne DAR using instrument simulators. Lebsock et al. (2015) specifically examine the potential for sounding water vapor within boundary layer clouds. They report an expected precision in CWV of 0.5–2 kgm^−2^ (~ 2–8% in the subtropics) with biases not exceeding 0.25 kgm^−2^. They find that profiling uncertainties are scene-dependent, ranging between 1 and 3 gm^−3^ at a range resolution of 500 m. Millan et al. (2016) apply an instrument simulator to global observations from CloudSat. They report precision in CWV of 0.3 kgm^−2^ with biases rarely exceeding 2.6 kgm^−2^ (10% of the global mean CWV). These larger biases are only found in heavily precipitating scenarios. They report an average single-bin precision of 89% with biases generally lower than 38% above 3 km and 77% near the surface.

Table 2 provides proposed observational requirements for a space-borne DAR. The DAR provides both profiling and CWV capabilities. The premise for the profiling requirements is that a DAR provides in-cloud profiling with the precision and resolution of a state-of-the-art infrared sounder, thereby extending water vapor profiling to all cloud scenarios. The requirements for the CWV observation are based on the current precision of passive microwave observations at very low spatial resolution over water surfaces only. DAR could advance the science possible with CWV observations by substantially increasing the spatial resolution (due to the high frequency employed) and extend sampling capability to all surface types thereby enabling quantification of the spatial variance of water vapor on small scales.

**Table 2:**
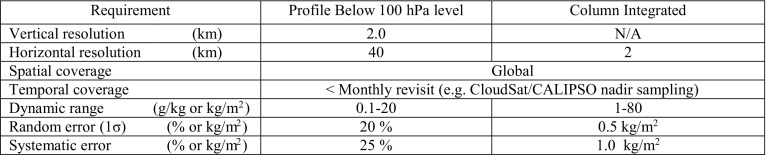
Proposed space-borne water vapor DAR observational requirements for profiling and column-integrated water vapor (CWV) measurements

Requirements for profiling mirror those of the Atmospheric InfraRed Sounder (AIRS; Auman et al. 2003), which samples the clear-sky water vapor, while those for precision of the CWV follow from the advanced microwave scanning radiometer (AMSR) for Earth observing systems. Expected precision of a DAR should exceed this required precision, possibly achieving 0.1 kg/m^2^ (i.e., Millan et al. 2016). The profiling capability would thus extend the clear-sky capability of state of the art infrared sounding into cloudy atmospheres. The column-integrated observations would provide an order of magnitude increase in spatial resolution over passive microwave and add capability over all surface types. Systematic error estimates are derived from the study of Millan et al. (2016)

### Technology Readiness

DAR is a newly emerging technology. There have only been initial observations of surface pressure using a 60 GHz DAR (Lawrence et al. 2011). A prototype water vapor DAR transceiver is currently being built at the Jet Propulsion Laboratory under the NASA Earth Science Technology Office’s Advanced Component Technology (ACT) and Instrument Incubator Program (IIP). Due to restrictions on radio-frequency transmission surrounding the 183 GHz line, the prototype instrument is being developed for the 167–174.8 GHz band, which is currently reserved for but unused for communications systems. This tunable, all-solid-state G-Band transceiver leverages recent innovations in extremely high frequency radar system architecture, Schottky diode frequency-multiplier power handling, and III-V semiconductor amplifiers. The transceiver’s TRL began at 2 (technology concept) and is currently at 4 (component/subsystem validation in laboratory environment) as of 2017. For space-flight, TRL-6 (system/subsystem model or prototyping demonstration in a relevant end-to-end environment) must be achieved. Additional investments in system design and integration will be required to increase the TRL of the G-Band DAR to 6 for both airborne and space-borne applications. In particular, maximum transmit power is currently in the 0.5 W range which is suitable for close range aircraft observations but must rise by 2 orders of magnitude to enable a satellite DAR. Such increases in transmit power are not manifestly infeasible in the near future using vacuum electronic power sources like those employed by current space-borne radars (e.g., CloudSat).

## Microwave Occultation

Based on the established and meanwhile widely successful technique of Global Navigation Satellite System (GNSS) radio occultation (RO), using the refraction of decimeter wave L-band signals near 1.2 and 1.6 GHz received at low-Earth orbit (LEO) satellites, temperature and humidity profiles in the troposphere can be retrieved only by cousing a priori temperature and/or humidity profile information (see Pincus et al. 2017, for a brief RO introduction in this special issue; for detailed review see, e.g., Kursinki et al. 1997; Anthes 2011; Steiner et al. 2011).

**Fig. 6:**
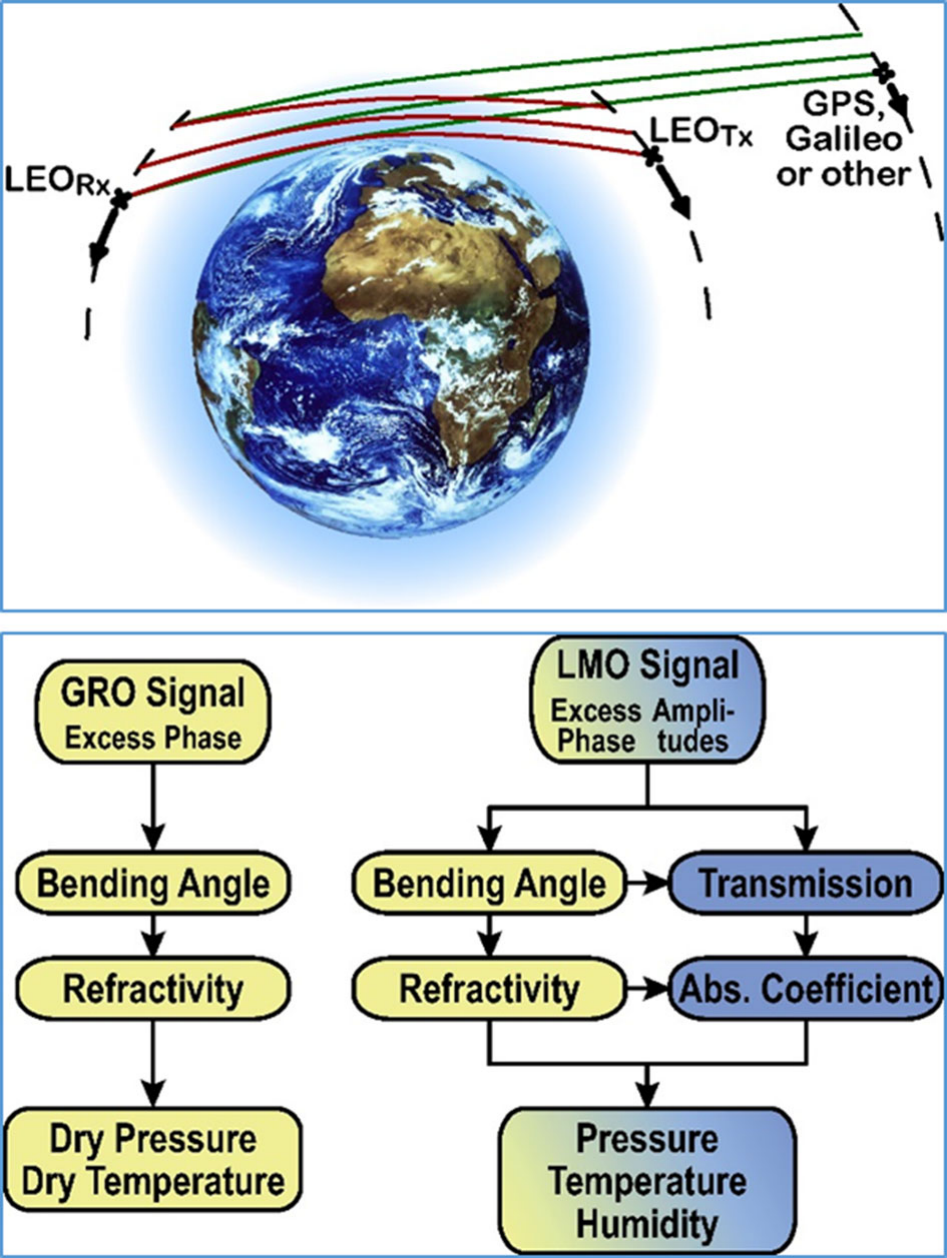
Schematic view of the LMO (red signal paths) and GRO (GNSS RO, with GPS and Galileo systems named as example; green signal paths) occultation event geometry (top panel), and schematic view of the retrieval processing chain for LMO (right) and GRO (left) data (bottom panel). *Source* Schweitzer et al. (2011)

As an emerging technique advanced from RO, LEO–LEO microwave occultation LMO), using centimeter and millimeter wave signals between LEO transmitter and receiver satellites, exploits both refraction and absorption (water vapor) of signals in the X/K band within 8–23 GHz to overcome RO’s temperature-humidity ambiguity in the troposphere. Figure 6 illustrates the LMO measurement technique compared to RO (for detailed LMO introduction see Kursinski et al. 2002, 2009; Kirchengast and Hoeg 2004; Schweitzer et al. 2011; for a recent review see Liu et al. 2017).

The LMO technique thus enables to retrieve pressure, temperature, and humidity profiles without a priori background information. It can also provide accurate profiling of these key thermodynamic state variables higher up over the full stratosphere, including water vapor if using also absorption signals near the 183 GHz water vapor absorption line, due to the insensitivity of the higher signal frequencies of LMO to ionospheric influences. Furthermore, stratospheric ozone profiles can be retrieved if additional absorption signals near the 195 GHz ozone absorption line are used. Liquid water and ice cloud properties as well as turbulence strength can be retrieved as by-products.

Since the late 1990s a series of LMO-related proposals and associated development work have been pursued, mainly in USA and Europe, which is reviewed by Liu et al. (2017). The current USA-led and European-led LMO mission concepts pursued toward implementation are ATOMMS—Active Temperature, Ozone, and Moisture Microwave Spectrometer (Kursinski et al. 2009, 2012) and the LMO component of ACCURATE—Atmospheric Climate and Chemistry in the UTLS Region And climate Trends Explorer (Kirchengast and Schweitzer 2011; Schweitzer et al. 2011). The unique potential of LMO strongly suggests that this ongoing work toward demonstration missions should lead to launches of such first missions in the 2020–2022 timeframe.

### Measurement Approach

The LMO measurement approach exploits the refraction and absorption of centimeter wave and optionally millimeter wave signals (within X/K band 8–23 GHz and optionally within 178–196 GHz) by performing accurate geodetic tracking measurements of the complex signal (phase and amplitude) at each chosen frequency during LEO-to-LEO occultation events, as illustrated in Fig. 6 (top panel) compared to GNSS RO. The atmospheric excess phase path due to refraction, relative to straight-line phase path in vacuum, and the amplitude attenuation due to water vapor (and optionally ozone) absorption, are derived from the raw tracking data of phase and amplitude and are the basis for subsequent retrieval of bending angle/refractivity profiles and transmission/absorption coefficient profiles, respectively, as schematically shown in Fig. 6 (lower panel) compared to GNSS RO. The well proven GNSS RO approach (Kursinski et al. 1997; Anthes 2011; Pincus et al. 2017) is shown for comparison with facilitate understanding that LMO works very similar in terms of geometry (with the transmitters in LEO instead of GNSS orbits near 20,000 km altitude) and fully shares the refractometric part of measurement principle.

The core algorithm steps for RO are: (1) complementing the “refractive” Abel transform for refractivity profile retrieval from bending angle profiles (Kursinski et al. 1997), an “absorptive” Abel transform yields differential absorption coefficient profiles retrieval from differential transmission (amplitude attenuation) profiles obtained from transmission differences between adjacent signal frequencies to eliminate broadband effects (Kursinski et al. 2002); (2) The joint availability of refractivity and differential absorption coefficient profiles then allows to unambiguously retrieve pressure, temperature, and humidity profiles by an optimal estimation scheme (Schweitzer et al. 2011). A detailed description of the LMO measurement approach and detailed algorithm descriptions of the steps in the retrieval processing chain are provided by Kursinsiki et al. (2002), Gorbunov and Kirchengast (2007), and Schweitzer et al. (2011).

### Measurement Capabilities

In order to summarize the mission performance and information provided by LMO, and in this way concisely indicating LMO’s capabilities and limitations, Table 3 summarizes observational requirements of a typical LMO mission concept (ATOMMS) based on the review by Liu et al. (2017). The measurement requirements are derived from a combination of flow down requirements needed to advance global climate monitoring and numerical weather prediction (Liu et al. 2017) as well from physical measurement limitations. Achieving this required performance for temperature and humidity profiling by suitable LMO instrumentation will at the same time ensure adequate performance also for the other variables such as pressure and other by-products. Four to five signals at X/K band frequencies, and optionally two to five signals within 178–196 GHz (max. three if without ozone capability), ensure the needed information content is provided for the retrieval.

**Table 3:**
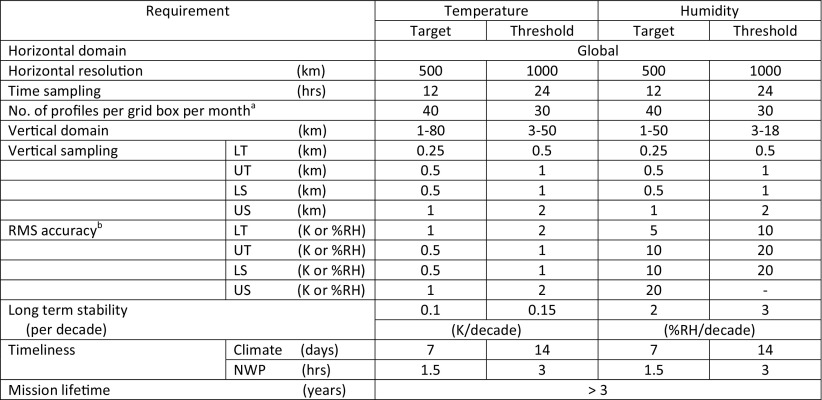
LMO observational requirements (based on ATOMMS requirements table of Liu et al. 2017)

^a^No. of profiles to be fulfilled in global average by all grid boxes but also any individual grid box shall receive at least 80% of this number. Grid box is here defined as square of the horizontal sampling requirement [box of size Horiz. sampling (km) × Horiz. sampling (km)] or any box of equivalent size with at least 500 km length of its smaller dimension

^b^Understood to be the accuracy for an individual occultation event over the required vertical domain (in the LT from Top of BL upward) at a vertical resolution consistent with the required sampling [i.e., a resolution of 2 × vertical sampling (km)]

Regarding the density of coverage in space and time with occultation events, the number of transmitter (Tx) and receiver (Rx) satellites is key. Generally designing Tx and Rx satellites into counter-rotating orbits for optimal event geometry (Fig. 6), initial demonstration missions typically aim at small 2 Tx and 2 Rx constellations (Kirchengast and Hoeg 2004; Kirchengast and Schweitzer 2011), leading to about 240 events per day, comparable to the initial GNSS RO demonstration mission CHAMP, Challenging Mini-Satellite Payload (Wickert et al. 2001). Subsequent operational LMO missions will intend to provide a much higher number, well meeting the sampling requirements. For example, a 6 Tx and 6 Rx constellation can provide about 3800 events per day (Kirchengast and Schweitzer 2011), more than the successful COSMIC six-satellite RO constellation (Anthes et al. 2008).

**Fig. 7:**
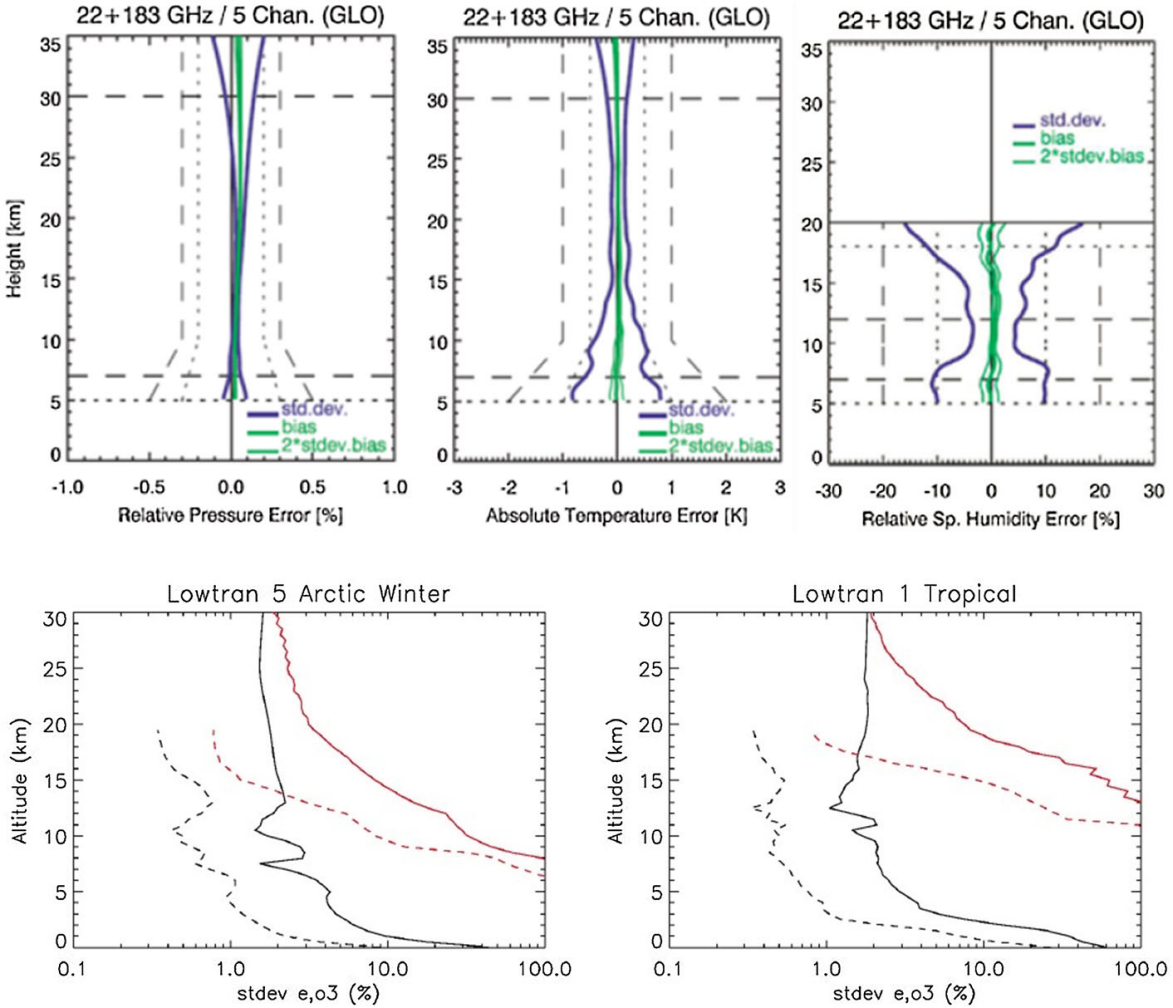
Retrieval performance estimated for pressure, temperature and specific humidity profiles for LMO, using three K band channels and a 179/182-GHz channel pair [top panels; *Source* Schweitzer et al. (2011)], and retrieval performance estimated for water vapor (black) and ozone (red) profiles for LMO (solid) and aircraft occultations (dashed) in Arctic winter (left) and tropical (right) conditions [bottom panels; *Source* Kursinski et al. (2016); E.R. Kursinski, pers. communications (2016)]. Schweitzer et al. (2011) assessed the performance by employing end-to-end simulations of a full-day ensemble of a few hundred LMO events, using realistic orbits, ray tracing through an atmospheric NWP analysis field, and observational error models for obtaining simulated LMO observations. An atmospheric profiles retrieval processing chain was then run and its results compared to the “true” NWP analysis profiles at the LMO event locations, for estimating performance statistics such as those shown in the top panels. Kursinski et al. (2016) used a somewhat simpler but similar approach, with forward modeling toward reasonably realistic simulated observations, separately for LEO-to-LEO and aircraft-to-aircraft geometries, and then applying atmospheric retrieval error estimation to obtain results such as shown in the bottom panels

A range of feasibility studies and technology projects for LMO instruments, and on-ground demonstration experiments, have evidenced that also the required accuracy and stability is achievable (ESA 2004; Kursinski et al. 2012; Liu et al. 2017, and references therein). Figure 7 illustrates example performance results for pressure, temperature, and humidity profiling from quasi-realistic end-to-end simulations, accounting for the effects of instrumental errors, clouds, and scintillation noise from turbulence (Schweitzer et al. 2011; Kursinski et al. 2009). The profiles are generally found unbiased and within target accuracy requirements. The main limitation regarding lower troposphere water vapor profiling is degraded accuracy and poor spatial resolution in the atmospheric boundary layer similar to RO. The strength of the technique is in the free troposphere down to the top of the boundary layer, which itself can be detected with high accuracy (Anthes 2011; Ho et al. 2015) and allows other active and passive remote measurements to optimize sensitivity for the lower troposphere.

### Technology Readiness

A series of technological feasibility studies and technology development projects for LMO instrumentation and related ground-based demonstration campaigns (ESA 2004; Kursinski et al. 2009, 2012; Kirchengast and Schweitzer 2011; Liu et al. 2017; and references therein) have prepared the basic technological readiness for now working toward LMO demonstration missions targeted for launch in the 2020–2022 timeframe. This applies in particular to the USA-led ATOMMS (Kursinski et al. 2009, 2012) and the Europe-led ACCURATE/LMO mission concepts (Kirchengast and Schweitzer 2011; Liu et al. 2017), where in various relevant studies also associated data processing chains have been developed to good maturity.

In China, a LMO pre-study has been carried out at the National Space Science Center (NSSC Beijing), which included design and performance analysis studies and prototype transmitter and receiver development (Liu et al. 2017). A pair of LMO transmitter and receiver has been manufactured and a related ground-based experiment is scheduled for later in 2017. Recent advances in the development of small satellites in the Microsat/Nanosat class now increasingly provide an economical way to realize LMO missions. This avenue is therefore pursued next as summarized by Liu et al. (2017).

In view of the promise of highly cost-effective potential LMO missions in the Microsat/Nanosat class, the most relevant next technology advancements required are those toward LMO instruments miniaturization, in order to become compliant with a Microsat/Nanosat-type LMO demonstration mission approach. Working in this direction, a recently commenced European–Chinese cooperation initiative toward an ACCURATE-1 LMO mission focuses mainly on initial X/K band technology and science demonstration, while a USA initiative toward an ATOMMS-1 mission intends both X/K band and 178–196 GHz band demonstrations.

Complementary to the activities related to LMO hardware, also further advancements of LMO data processing are required, as well as further demonstration campaigns. Hopefully, in the future, the LMO technique will combine with the growing observation density of GNSS RO to jointly profile the atmospheric thermodynamic state, and in particular water vapor in the (free) troposphere, with steadily increasing accuracy and resolution.

## Hyperspectral Microwave

Current down-looking passive microwave humidity sensors are limited to only a few spectral channels, typically less than ten. This is comparable to the situation of infrared sensors before the arrival of hyperspectral sensors such as the Atmospheric Infrared Sounder (AIRS) and IASI, both summarized in a recent intercalibration article by Wang et al. (2015). Despite their spectral limitations, microwave sensors play a big role in operational meteorology, because of their key advantage of being only weakly affect by clouds.

Recently, a new detector technology has emerged, Kinetic Inductance Detectors (KIDs) (Day et al. 2003; Doyle et al. 2008; Mauskopf et al. 2014; Baselmans et al. 2008; Bueno et al. 2014; Griffin et al. 2015). Each detector is a superconducting resonator circuit element, built by lithography, and cooled to a sub-Kelvin temperature, i.e. at a fraction of 1° K. These detector circuit elements present an inductive load to a superconducting microwave stripline, with each detector element tuned to resonate at a slightly different frequency. Incident photons on a detector break superconducting Cooper pairs, which changes the population of quasi-particles in the superconducting film. This alters the kinetic inductance of the film, with the effect of modifying the resonant frequency of the circuit. This results in a shift of detector frequency, amplitude and phase, proportional to the incident photon flux, and this is the detection signal.

The key advantages of this new technology are that detectors are relatively straightforward to produce in large arrays, and they are very sensitive, with photon-noise limited performance demonstrated for a variety of applications (Griffin et al. 2015). KIDs also allow a high multiplexing ratio, such that up to 1000 pixels can be read out on one readout line, which greatly simplifies instrument design. Recent developments have demonstrated the ability to implement KIDs as on-chip spectrometers (Shirokoff et al. 2014; Endo et al. 2012). In this implementation, a broadband antenna feeds a filterbank, where each narrow-band filter feeds and individual KID. We propose to optimize such a configuration (channel optimization) for applications to meteorology and climatology. Such a configuration allows photon-noise-limited sensitivity in the frequency region of approximately 100–1000 GHz, with individual spectral channel resolution (ν/Δν) of approximately 800. Studies are currently in progress to investigate alternate materials to demonstrate response in the region 50–60 GHz.

**Fig. 8:**
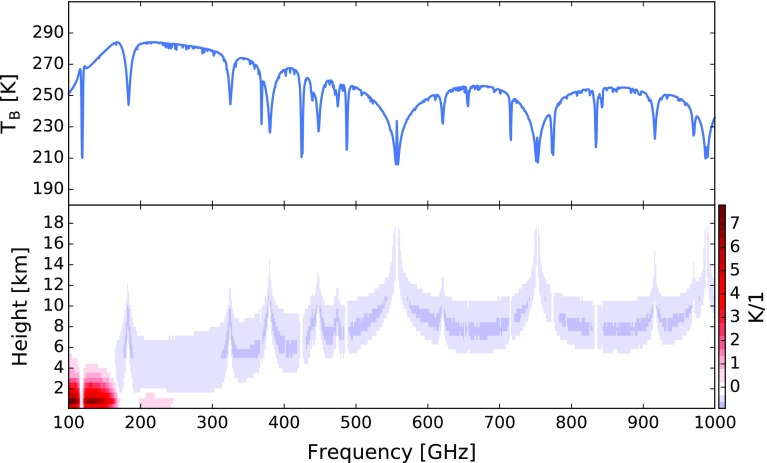
Simulated brightness temperature spectrum for a tropical atmosphere (top) and associated humidity Jacobian (bottom). The Jacobian roughly shows the brightness temperature change in Kelvin for a local doubling of the water vapor volume mixing ratio on a 0.5 km vertical grid. Blue colors indicate that the Jacobian is negative, which is typically the case for frequencies above approximately 170 GHz

Combined, the large array capability and high sensitivity can be exploited to build a hyperspectral microwave detector. The high sensitivity requirement here is perhaps less obvious than the high pixel number one. But according to the radiometer formula

$$ \Delta T = \frac{{T_{s} }}{{\sqrt {\Delta \nu \Delta t} }}, $$

the noise of a spectral channel ($\Delta T$) is proportional to the spectral bandwidth (Δν) to the power of minus one half, with the consequence that hyperspectral instruments with conventional detectors would be quite noisy. (The other two parameters in the above equation are a system-specific constant (*T*_*s*_) and the integration time (Δt).) The measurement approach of the proposed instrument is to passively measure a large part of the millimeter/submillimeter spectral range with a moderately high spectral resolution. The initial instrument concept studied below by retrieval simulations had 2303 channels between 100 and 1000 GHz. The simulated brightness temperature spectrum for a tropical atmosphere is shown in the top plot of Fig. 8. The bottom plot of the same figure shows the humidity Jacobian, i.e., the derivative of the spectrum with respect to fractional changes in the water vapor volume mixing ratio (VMR) at different altitudes. The simulation was performed with the Atmospheric Radiative Transfer Simulator (ARTS) (Eriksson et al. 2011). The data analysis for such an instrument would benefit from various laboratory studies for spectroscopic parameters in the submillimeter spectral range that were carried out in the last years, for example in the context of planned limb sounding missions (Perrin et al. 2005; Verdes et al. 2005).

### Measurement Capabilities

We have studied the capabilities of a hypothetical KID-based hyperspectral microwave sensor by optimal estimation (Rodgers 1990) retrieval simulations. As a reference, we also used the same retrieval setup on an idealized hyperspectral infrared sensor, emulating a future generation of IASI (the spectral resolution is taken from the current IASI instrument, but the noise is lower to allow for technical development and give a fairer comparison). The retrieval setup is summarized in Table 4. It closely follows the study by Schneider and Hase (2011). Besides temperature and water vapor, other relevant trace gases that significantly affect the spectrum are also included but currently not retrieved. For the KID microwave sensor this is mainly oxygen, but also ozone is quite important, because its lines are so ubiquitous (e.g., John and Buehler 2004). For simplicity, the KID microwave instrument was assumed to have regularly spaced channels with ν/Δν = 1000, resulting in more than 2000 channels. But the number of channels could be reduced significantly without performance degradation if channels are chosen intelligently, since information content is not distributed equally. Initial tests show that approximately 100 channels would be enough in practice.

**Table 4:** Summary of retrieval simulation setup

Infrared	8534 Channels, 19–83 THz
Sensor	NeΔT 0.1–0.2 K
Microwave	2303 channels, 100–1000 GHz
Sensor	NeΔT 0.007–0.024 K
Retrieved	Temperature, retrieved in Kelvin
Species	Water vapor, retrieved in ln(VMR) coordinates
Water vapor a priori covariance matrix	Depending on altitude, mimicking Schneider and Hase (2011)
	Standard deviation 1–0.25 in ln(VMR) coordinates
	Correlation length 2.5–10 km
Atmosphere	FASCOD tropical atmosphere (Anderson et al. 1986)
Surface emissivity	Microwave: 0.6; infrared: 1.0

NeΔT indicates the instrument noise, which was assumed to vary across the band, based on typical instrument behavior, with the range indicated

*VMR* volume mixing ratio

**Fig. 9:**
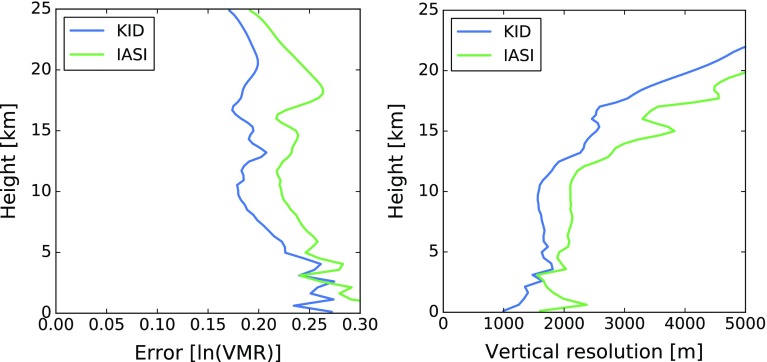
Simulated water vapor retrieval error profiles for microwave and infrared instrument (left) and vertical resolution of the measurements (right). An ln(VMR) error of 0.1 roughly corresponds to a relative error of 10% on the VMR

The left plot in Fig. 9 shows the retrieval error for water vapor on a 0.5 km retrieval grid for both sensors. One caveat is that surface emissivity here was assumed to be known perfectly, whereas in reality there is some uncertainty on this knowledge, especially over desert and arid surfaces. The very small retrieval errors close to the surface for both instruments therefore are overoptimistic. Overall, according to this simulation the KID microwave sensor would perform significantly better than the infrared sensor in the troposphere. The KID microwave sensor noise presented here is based on the assumption of a pushbroom geometry. An alternative (preferred) KID-based solution is a scanning geometry that uses the array only for the detection of different frequencies, not for different spatial pixels. For a sensor scanning over 100 spatial pixels, instead of measuring them simultaneously, the noise would increase by a factor of 10. Simulations were also carried out with the noise increased by a factor of 10, and as expected they show higher retrieval errors and a poorer vertical resolution, but still competitive with the IASI simulation.

Of course, a crucial question is also the vertical resolution of the measurement. The right plot in Fig. 9 shows the vertical resolution of the retrieved water vapor profiles for both sensors, computed as the full width at half maximum of the averaging kernel functions for retrieval on a 0.5 km vertical grid. Even here the KID sensor performs better than the infrared sensor according to our preliminary simulations. Since the inherent vertical resolution of the measurement is poorer than the chosen 0.5 km retrieval grid, part of the total error in the left plot is due to smoothing error. When retrieval grid resolution is reduced to 1 km in an alternative retrieval setup, retrieval error is reduced by a factor of 2. Comparing these preliminary KID sensor simulations to the expected enhanced performance of IASI next generation (IASI-NG) still shows promise. IASI-NG will have 16,923 spectral channels covering roughly the same bandwidth as IASI, providing twice the spectral resolution (0.25 cm^−1^) and hence higher vertical resolution resulting from narrower weighting functions (Crevoisier et al. 2014). A major advancement of IASI-NG over IASI is the 2× improvement in radiometric noise, leading to reduction in retrieved water vapor uncertainty by ~ 3–5%, although these improvements are realized between 800 and 200 hPa (Crevoisier et al. 2014). The vertical resolution and accuracy of IASI-NG water vapor retrievals below 800 hPa will remain approximately the same as IASI, thereby maintaining the relevance of preliminary instrument performance simulations for KID sensors presented here and detailed space-based water vapor DIAL simulations presented in Gerard et al. (2004).

These performance figures depend strongly on the spectral resolution as well as the assumed instrument noise (NeΔT in Table 4). It is therefore quite possible that a future cooled infrared instrument would achieve even better performance. However, a key advantage of the KID microwave sensor is that it would be able to see through most of the clouds that are opaque to the infrared sensor. Additionally, it would provide information on the cloud hydrometeors. The scientific background for that measurement is discussed in Buehler et al. (2012) and Jiménez et al. (2007). Retrieval simulations for cloud parameters in the submillimeter spectral range so far have been done only for sensors with a small number of channels. Exploiting a hyperspectral sensor for this purpose needs faster radiative transfer algorithms, a development that is already ongoing. It is likely that there will be a large benefit, mostly because measuring the full spectrum should allow for a much better separation of the cloud signal from the water vapor signal, so that humidity and clouds really could be retrieved independently, with only little error correlation. However, this is yet to be demonstrated with explicit retrieval simulations.

In summary, we have demonstrated that a KID sensor would deliver high resolution and precision water vapor profiles, comparable or better to a hyperspectral infrared sensor, and with much better global coverage, since it would work in most of the infrared-cloudy areas, i.e., through thin clouds with water paths below a few grams per square meter. This is a significant advantage for operational meteorology, since cloudy areas likely have a strong impact on forecast skill. It also is a significant advantage for climatology, because the clear-sky sampling of infrared sensors introduces a humidity dry bias (John et al. 2011). The capabilities of such a sensor to deliver hydrometeor profiles and water vapor profiles in areas with thick clouds still have to be demonstrated, but are likely to constitute a significant advancement over currently planned submillimeter sensors that are based on conventional technology. Threshold requirements for profiling water vapor in clear air for operational meteorology and climate studies would be similar to those of infrared sounders such as AIRS and IASI.

### Technology Readiness

Kinetic inductance detectors are a new technology, first proposed by Day et al. (2003). But since then they have progressed rapidly. A recently completed European Union-funded project, SPACEKIDS (Griffin et al. 2015), delivered two laboratory prototype instruments, one of which was optimized for observing the Earth’s atmosphere, a high radiative background case compared to typical applications in astronomy. Currently, array sizes of up to 1000 pixels can be realized in the laboratory with a single readout line and electronics set. In addition, full filterbank implementations have been demonstrated in a laboratory environment (Shirokoff et al. 2014; Endo et al. 2012).

The focal plane would need to be cooled to ~ 300 mK, with an operational design lifetime goal of around 7 years. This requires high reliability mechanical cryocoolers, attached to a ^3^He sorption cooling system. The ^3^He system would be based upon proven heritage from Herschel PACS and SPIRE (Griffin et al. 2008) instruments, with the possibility of continuous 300 mK cooling, recently developed by Cardiff University, in collaboration with Chase Research.

The Planck JT compressors were supplied by EADS Astrium, based upon a licensed design from RAL, the compressors are first generation linear motor reciprocating mechanisms originally developed for Stirling cycle coolers which have now amassed around 15 years of in-orbit operational life between 24 mechanisms. The 4 K cooler requires pre-cooling to temperatures below the inversion temperature for helium (about 27 K). In practice, the lower the pre-cooling temperature (within limits), the better the cooling power. The RAL two-stage 15 K cooler has been in development for many years and is now able to deliver around 250 mW at 15.4 K and a no load base temperature of 9.8 K. The compressor input power under these conditions would be around 135 W. This could provide the basis of an excellent pre-cooler for our instrument. In summary, overall the technology is at a readiness level where the logical next step would be to build an airborne prototype instrument, or a small demonstration satellite mission. The remaining challenge is foreseen in the design of radiators with sufficient cooling power to suit the higher infrared radiation load from Earth (compared to applications for astronomy) that a meteorological mission in low-Earth orbit would be exposed to.

## Synergies of Observing Systems

As the previous subsections have shown, promising technologies for improved water vapor profiling are emerging and may be operationally applicable within the time frame of a decade. However, no single remote sensing technology will ever deliver simultaneously full height coverage of water vapor in a highly, temporally and spatially resolving manner in all atmospheric conditions, which would be highly relevant for understanding and modeling atmospheric circulation patterns on a wide variety of scales. Profiling lidar systems are severely hampered by thick clouds, whereas future DAR systems will rely on the presence of backscattering particles within clouds. Passive remote sensing systems in the microwave region can retrieve water vapor in the presence of clouds, are, however, hampered by surface emission and rather low vertical resolution. Active limb sounding by LMO will feature high vertical resolution, no surface interference, and high accuracy and long-term stability but deliver comparatively sparse horizontal coverage and resolution unless large microsatellite constellations are deployed. Passive infrared spectrometers can accomplish a higher horizontal resolution, but are again disturbed by the presence of clouds.

Clearly, this calls for an optimized synergy of the different observation systems, complementing their respective strengths in different situations. Such methods have been developed successfully for classifying cloud type as well as for retrieving microphysical quantities from Cloudsat/CALIPSO cloud radar and lidar observations (Ceccaldi et al. 2013; Stein et al. 2011) but also using the Cloudnet instrument suite (Illingworth et al. 2007) from surface-based remote sensing observations.

### Value of Ground-Based Profiling

The bulk of ground-based atmospheric profiling has historically been undertaken by routine upper-air-soundings, but worldwide the density of this network is under pressure. Clearly, upper-air-soundings state benchmark measurements when it comes to profiling the atmosphere in terms of humidity, temperature and winds. However, carrying out upper-air-soundings on a routine basis presents enormous cost and labor factors. Also, launches are typically on a 12-h basis, so that the diurnal cycle of the boundary layer development and other short-lived weather events will not be captured. Certainly, such temporal developments are crucial for the initialization and evaluation of atmospheric models. Additionally, single ascents need approx. 1 h to pass through the troposphere and also underlie wind drifts effects. Another drawback concerning upper-air-soundings is that no methods have been established to measure cloud macro- and microphysical properties on an operational basis.

Ground-based remote sensing has an enormous potential for accurately profiling the lower troposphere with high vertical and temporal resolution as shown by Wulfmeyer et al. (2015). It has been known for some time that passive microwave radiometers (MWR) offer the potential of filling the gap in retrieving the atmospheric thermodynamic state (Westwater 1997), i.e., the distribution of temperature, humidity and liquid water, in a quasi-continuous and instantaneous manner. Additionally ground-based infrared spectrometers, such as the commercially available atmospheric emitted radiance interferometer (AERI) instrument (Knuteson et al. 2004) also show high potential for retrieving temperature and humidity profiles from the surface (Turner and Löhnert 2014)—in addition to cloud and aerosol parameters. While technical developments of the last decades have led to passive systems, which can operationally profile the thermodynamic structure of the atmosphere, their vertical resolution is rather low showing the best retrieval performance in the lower part of the boundary layer. Typical vertical resolutions are on the order of 50 to 500 m in the lower troposphere. Active lidar systems (employing either DIAL or Raman technology) are able to detect sharp vertical gradients in humidity and/or temperature with much smaller uncertainties in general. Many ground-based lidar systems, however, do not provide full coverage of the troposphere, can show daytime-dependent noise characteristics and are not yet at a technical development stage to be called fully mature for 24/7 unattended operation. Exceptions to the aforementioned generalization, among others not cited here, are the fully operational DOE ARM Raman lidar (Turner et al. 2016), the NCAR field deployable micropulse DIAL (Spuler et al. 2015; Weckwerth et al. 2016; Nehrir et al. 2011a, b, 2012), the Cloud Observing Radar and Lidar at the Barbados cloud observatory (Stevens et al. 2016), and the Raman Lidar for Meteorological Observations at the MeteoSwiss Regional Center of Payerne (Brocard et al. 2013), all of which exhibit varying levels (spatial and temporal) of daytime dependent noise characteristics. Still, promising developments will enable many more operational ground-based lidars to come online within the next decade with improved capabilities for measurements in the mid-lower troposphere.

Ground-based remote sensing profiling can be a benchmark for accurately and continuously profiling the lower troposphere if synergetic methods are applied to exploit the complementary potential of the different measurement systems. Developments in this direction have just begun and require scientific expertise in different observation systems simultaneously as well as the employment of advanced retrieval methods solving the classical inverse problem of atmospheric remote sensing in multiple ways. Examples for such developments are given in the next section. Ebell et al. (2017) retrieve cloud, water vapor and temperature profiles simultaneously through synergy of ground-based active and passive remote sensing. Such observations are highly important for model evaluation, e.g., for the small-scale, cloud-resolving model ICON-LEM (Heinze et al. 2017) developed with the HD(CP)^2^ (High Definition Cloud and Precipitation for Climate Prediction) research initiative as well as for process studies on clouds and precipitation.

One demanding question to be resolved is to what extent local ground-based remote sensing observations could complement existing measurements within the global observation system. Here, the combination of existing satellite and ground-based profiling methods can lead to an increase in local information content (see next section) for the simultaneously observed atmospheric column. However, research is also required to what extent a local observation with continuous and temporally highly resolved profile information can improve satellite observations within a certain radius of influence.

### Examples of Synergetic Applications

In the following two examples of synergetic retrieval applications for atmospheric thermodynamic profiling are given. These are intended to show the potential of variational methods for optimally exploiting the information content contained in the respective observations resulting in a retrieval with an improved performance with respect to overall uncertainty and information content. Note, an objective way to analyze the information content of variational retrievals is to evaluate the number of degrees of freedom (DOF) for signal for the retrieved profile; i.e., the number of independent levels of humidity that can be determined.

#### Combining Lidar and Microwave Radiometer

**Fig. 10:**
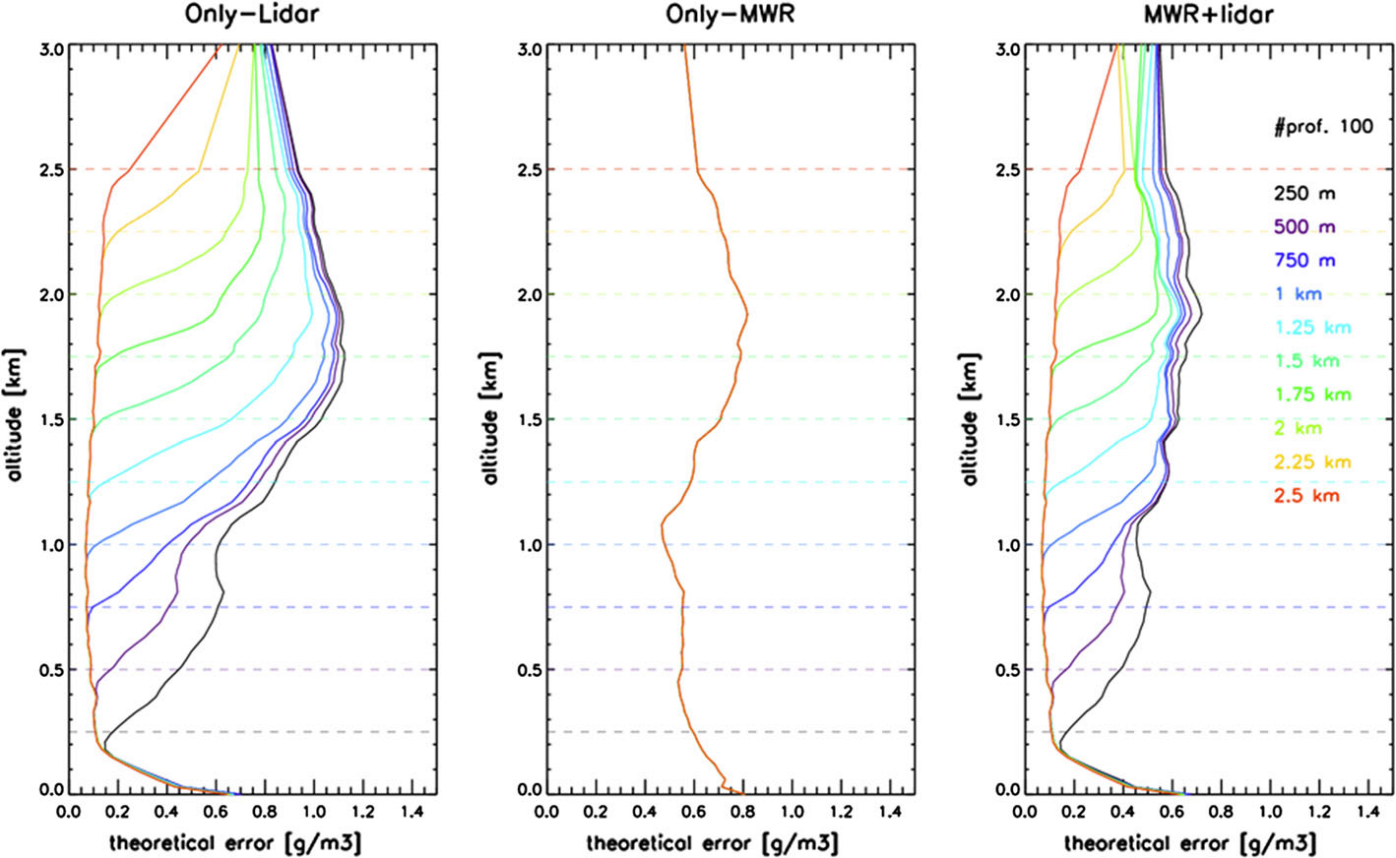
Mean uncertainty of 100 clear-sky cases of absolute humidity retrieval during HOPE. Left: Water vapor lidar only, Center: MWR only, Right: Combination of both lidar and MWR. The dashed horizontal lines indicate levels of full hypothetical lidar attenuation, i.e., due to clouds. The corresponding colored lines (bold) show the uncertainty in case the lidar provides no signal above the dashed line

During HOPE (HD(CP)^2^ Observational Prototype Experiment, Macke et al. 2017), water vapor Raman lidar and microwave radiometer observations were carried out simultaneously at JOYCE (Jülich Observatory Cloud Evoultion, Löhnert et al. 2015) for profiling water vapor profiles. A variational retrieval was applied (Barrera-Verdejo et al. 2016) to optimally exploit both observation systems leading to the uncertainty estimates of the profile shown in Fig. 10. Lidar-only retrievals that are carried out in cloud-free conditions are, as expected from the physical basis of the problem, significantly more accurate than the MWR-only retrieval in terms of standard uncertainty (compare Fig. 10 left and center panels, red lines). The lidar signal is assumed to be attenuated at varying heights, i.e., due to clouds, the lidar-only retrieval uncertainty rapidly increases with height toward the a priori profile. However, the benefit of the synergy of both systems becomes clear in exactly these situations (Fig. 10 right panel). The retrieval uncertainty stays well below the lidar-only and MWR-only values throughout all heights, with the highest impact visible in the heights above 1 km (up to 0.3 gm^−3^). In these cases, the degrees of freedom for signal of the MWR (~ 2) are used to improve the profile above the height of full lidar extinction, where in the MWR-only case these DOF are used to retrieve the full profile leading to the observed large uncertainties on the order of 0.6 gm^−3^.

Ground-based MWR observations (HATPRO) can also be combined with highly spectrally resolved infrared observations (AERI) to improve the DOF of the humidity profile to be ~ 4 (Ebell et al. 2013). Here, the additional information is distributed throughout the whole atmospheric profile.

#### Combining Satellite and Ground-Based Observations

**Fig. 11:**
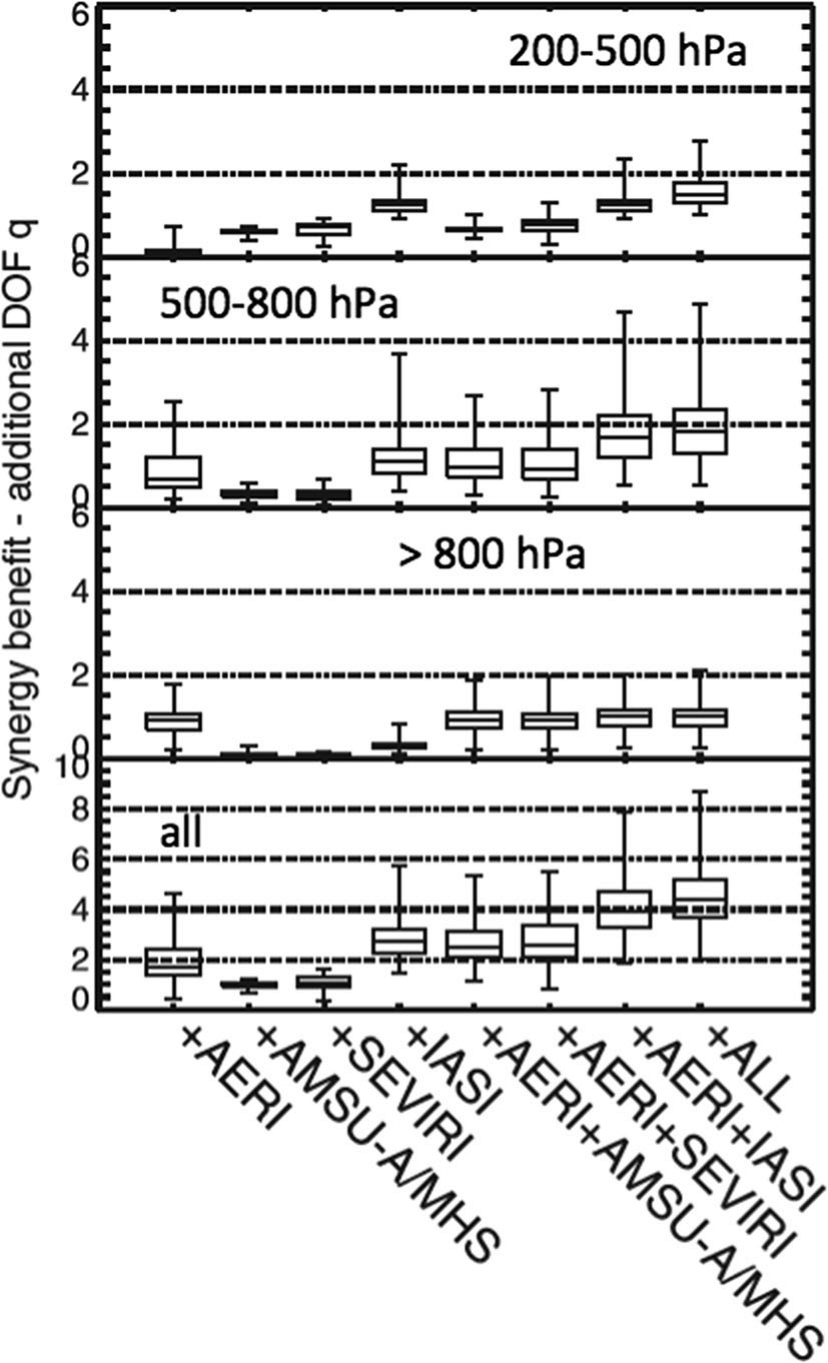
Synergy benefit for absolute humidity profile in terms of additional DOF compared to HATPRO-only retrieval. Median (line in box), 0.25 and 0.75 quantiles (box boundaries), and minimum and maximum values (whiskers) of the profile sample are shown. From Ebell et al. (2013)

Satellite sounders typically only provide little information in the boundary layer wherefore the combination with ground-based sensors proves beneficial (Fig. 11). Combining AMSU-A/MHS microwave satellite sensors with ground-based MWR is less beneficial than the combination with IASI, the latter being a satellite-based spectrally highly resolving infrared spectrometer. Compared to the HATPRO-only retrieval, the DOF increases by 2.5. The main advantage of adding SEVIRI (geostationary satellite data) to ground-based MWR observations is obtained between 200 and 500 hPa. In this study, the additional DOF is roughly equal to the sum of the DOF of a single instrument so one can conclude that the information content of the sensors is optimally combined. Combining all sensors used in this study results in the largest gain of information content.

### Future Perspectives

There is strong need for combining satellite and ground-based observations of wind, water vapor and clouds. The examples motivate that improvements in uncertainty reduction and information content gain are possible; however, the synergy even of existing observation systems is today far from optimally exploited. Physically consistent combinations of observations will become more important with the advent of advanced profiling systems, which will work under specific atmospheric conditions.

Passive instruments on geostationary satellites, but especially on current polar-orbiters are basically the “workhorses” for capturing the mid- and upper-tropospheric dynamic and thermodynamic environment (including clouds). It is imperative that the international atmospheric science community pursues the sustainment of such measurement systems in space (preferably with increased spectral resolution) in order to guarantee these vitally important data streams for global assimilation systems in the decades to come. In spite of their importance for large-scale flows, however, these sensors reveal limited retrieval performance in the boundary layer. Future satellite concepts could thus be comprised of a passive cross-track, high spectral resolution scanner in addition to a nadir pointing active system for vertically highly resolved profiles of water vapor in the column above the satellite track.

Feasibility studies of such a system could be carried out at ground-based remote sensing sites using manifold observations of the boundary layer (i.e., in critical regions of convective aggregation) through a campaign-based deployment of mobile and/or fixed ground-based profiling stations including Doppler lidar (BL winds), cloud radar (BL clouds), microwave radiometer (BL temperature and humidity) and DIAL and/or Raman (humidity profiles below clouds). A much more complete picture of the column could then be obtained by directly combining satellite and ground-based measurements within variational approaches. The representativeness for going from a point measurement to an area is an additional challenge, but could be resolved by overpasses (curtains) either from Cloudsat/CALIPSO or from dedicated aircraft (HALO, HIAPER, NASA DC-8 aircraft) observations. A complete picture of the region of interest would probably need to involve an advanced variational data assimilation scheme with these multiple types of sensors.

## Summary and Conclusions

Water vapor is the most dominant greenhouse gas and plays a vital role in both the climate and weather. Water vapor profile measurements with improved accuracy and vertical resolution would benefit many major research programs including the World Climate Research Programs (WCRP), the World Weather Research Program (WWRP), the Stratosphere-troposphere Processes and their Role in Climate (SPARC) program, the Network for the Detection of Atmospheric Composition Change (NDACC), and the Global Energy and Water Cycle Experiment (GEWEX) program. In addition to providing lower tropospheric profiles, the discussed measurement approaches can provide simultaneous high accuracy and high vertical resolution water vapor profiles in the UTLS that are needed to address both the chemical and radiative role of water vapor to changes in climate. Accurate, high vertical resolution water vapor profiles would also improve estimates of surface evaporation and surface fluxes of sensible and latent heat which are critical to numerous boundary layer processes important to both weather and climate, but also for constraining atmospheric heat budgets across scales. The recent NASA Weather Focus Area community workshop report (Zeng et al. 2015) calls for a continuing investment to improve satellite measurements of water vapor in the atmospheric boundary layer.

Current satellite water vapor products rely mainly on passive measurements of spectrally resolved microwave and infrared radiances at the top of the atmosphere, but such radiances are weighted toward the mid-troposphere and are not very sensitive to water vapor in the lower troposphere where it varies the most. The radiative kernel functions are broad and are unable to resolve either the marine boundary layer or the gradients in water vapor near the trade inversion, both of which are critical to processes that govern shallow cloud cover and convection initiation. Poor vertical resolution also mutes circulation patterns associated with the radiative response to large water vapor gradients. Passive infrared measurements have a vertical resolution of at best 1–2 km in the lower troposphere with relative errors of 10–20% (August et al. 2012). Microwave profilers can provide humidity measurements in regions of non-precipitating clouds; however, they can suffer from underlying biases resulting from uncertainties in the microwave surface emissivity. Tropospheric humidity retrievals from GNSS radio occultation show promise due to their favorable combination of high vertical resolution, accuracy, long-term stability, all-weather capability and global as well as local time coverage, but are limited in their horizontal resolution (especially near the surface) and by the need for a priori background temperature profiles required to retrieve humidity profiles.

Space-based water vapor lidars are ideal for high accuracy measurements of water vapor throughout the troposphere and lower stratosphere as they are self-calibrating and not prone to bias resulting from aerosol and cloud contamination. Furthermore, they can retrieve water vapor profiles during day and night, at all latitudes, through broken cloud fields and tenuous high altitude cirrus (with reduced spatial resolution), and during all seasons. In addition to profiles of water vapor, DIAL can provide high accuracy measurements of total precipitable water, cloud occurrence, cloud-top-height, and cloud water phase. Further, gradients in both water vapor and aerosol can be used to characterize the height of the (marine) boundary layer inversion. This capability is useful for studying vapor-cloud interactions, although to comprehensively address questions related to shallow cloud processes, DIAL observations should be augmented with observations of cloud optical depth, liquid water path, and droplet size from passive sensors, in addition to detection of drizzle and light precipitation from radar.

Airborne DIAL prototypes have been fielded over the past 20 years demonstrating key technologies and resulting science capabilities from active sensing platforms. A concept study was carried out by Gerard et al. (2004) to assess the performance of a space-based water vapor DIAL in relation to passive measurements such as IASI. The results of the study indicated that for a fixed error threshold, a water vapor DIAL would extend the water vapor measurement to a greater altitude as well as further down into the lower troposphere than IASI, would provide higher precision and accuracy in the lower troposphere and would have approximately twice the vertical resolution. The study also indicated that the water vapor measurements would extend from the lower stratosphere down to the surface which would have significant positive impacts in determining the background error covariance used in Numerical Weather Prediction (NWP) analysis, as well as for providing a stable absolute reference for calibrating passive sensors. Although the DIAL technique has been extensively demonstrated via airborne simulators and is also the most mature of the technologies described here, a comprehensive funding profile is still required to increase the technology readiness level of critical subsystems such as the laser, detectors, and filters, required for a space-based implementation.

Complementary to DIAL, the DAR technique provides profiles of water vapor within clouds. The DAR technique presented here samples the 183 GHz water vapor absorption line with two discrete RF channels, one that sits on the wing of the absorption line and one that is located away from the absorption line. Similar to the DIAL technique, the ratio of the radar reflectivity’s at the online and off-line frequencies is approximately proportional to the water vapor path. The DAR technique is a powerful new emerging technology in that it will provide profile measurements of water vapor within clouds at different altitudes, an area which no satellite or airborne sensor currently has the capability to retrieve high accuracy measurements. A combined DIAL and DAR payload will serve as a powerful measurement suite providing both clear air and in-cloud profiles of water vapor, in addition to column measurements from both systems.

An extension of GNSS RO, LMO utilizes centimeter and millimeter wave signals between LEO satellites in the X and K bands to retrieve pressure, temperature, and humidity profiles using an optimal estimation framework. Together with pressure and temperature retrievals, the LMO water vapor retrievals show promise in providing high accuracy global profiles throughout the troposphere and stratosphere in clear and cloudy conditions. A primary limitation of LMO is an inherently limited horizontal resolution of approximately 100–200 km along the sounding paths, which is thus not able to resolve fine-scale features in water vapor fields in the near-surface atmosphere. An LMO observing system coupled with nadir pointing active and passive systems, which provide high spatial resolution measurements in the lower troposphere, would extend the vertical coverage of water vapor profiles from the stratosphere down to the surface. The first LMO demonstration missions, ACCURATE-1 and ATOMMS-1, are currently under development and expected to launch in the 2020–2022 timeframe.

Hyperspectral passive microwave KID sensors, as discussed above, would extend the capabilities of current hyperspectral infrared sensors to cloudy areas. This all-sky capability and the wide measurement swath would be advantages even compared to active DIAL measurements, although accuracy and horizontal and vertical resolution would be inferior to DIAL. In terms of technological readiness, hyperspectral KID sensors are the least mature of the techniques discussed here, but currently developing rapidly. Laboratory systems have already been realized, and the next step will be airborne prototypes, which could be ready within the next 5 years. A top level system/subsystem TRL breakdown based on the NASA Earth Science Technology Office definitions is presented for each measurement approach in Table 5. The TRL breakdown is intended to summarize the relative matureness of each measurement approach in order to estimate the potential readiness date for a future space-borne mission, rather than to elucidate the exact level of readiness of each instrument(s) subsystem(s), which is required when transitioning from ground/airborne demonstrators to a space-borne implementation.

**Table 5:** System/subsystem technology readiness levels for the different airborne and space-based technologies presented here. For orientation, TRL 2 is a technology concept, TRL 4 is component/subsystem validation in a laboratory environment, TRL 6 is system/subsystem model or prototyping demonstration in a relevant end-to-end environment

Measurement approach	Aircraft TRL	Space-borne TRL	Comment
*Differential absorption lidar*			
Transmitter	9	4–5	Nd/YAG pump lasers developed for current space-based lidar missions (e.g., ADM Aeolus, EarthCARE, MERLIN, CALIPSO follow-on mission concept) can be adapted for use as a pump laser for a water vapor DIAL transmitter. TRL for DIAL single-frequency pulsed and continuous wave seed lasers reflect technologies developed under the LASE, WALES, HALO, and MERLIN programs
Receiver	9	5–6	TRL reflects use of highly sensitive single photon sensitive silicon and mercury cadmium telluride (HgCdTe) detectors. Telescope adapted from ADM Aeolus. Frequency agile filter not included in TRL assessment
*Differential absorption radar*			
Transmitter	4	2	TRL based on existing GaN W-band power amplifier and GaAs Schottky diode frequency multiplication to achieve > 0.5 W continuous transmit power at G-band
Receiver	4	4	TRL based on proven quasi-optical duplexer and antenna and mature back-end digital electronics
*LEO–LEO microwave occultation*			
ATOMMS airborne demonstrator	5	NA	ATOMMS hardware developed for demonstration on NASA WB-57 platform. TRL for ground-based (mountain top) operation is at 8
ACCURATE microsat transmitter and receiver	NA	5	TRL reflects that successful phase A studies have been conducted and that related space instrument transmitter and receiver breadboard simulators have been developed and validated via ground-based (lower troposphere) long-path experiments
*Hyperspectral microwave*			
KID sensor	3	3	The KID spectrometer system is operated under vacuum in a cryogenic environment. The only difference with a satellite-based implementation is the enhanced cosmic ray flux. TRL is at 3 for space-based implementation, as the full cooling chain (radiator design) for LEO needs to be demonstrated

In addition to profiles of water vapor, information on the origin of water vapor is also important. Stable naturally occurring isotopes of hydrogen (Deuterium–Oxygen, HDO) have been used in hydrology for decades to provide constraints on the origins of water. The ratio of HDO to H_2_O (expressed as δD) is a tracer for water origin and lifetime and consequently sensitive to many key processes of the hydrological cycle such as mixing of air masses, convection, transport and cloud detrainment (Galewsky et al. 2016; Worden et al. 2007). Measurements of this water isotopologue therefore add value to measurements of the main isotope alone. Global HDO datasets have become available from satellites such as IASI, Tropospheric Emissions spectrometer (TES) for sensitivity in the upper troposphere or SCanning Imaging Absorption SpectroMeter for Atmospheric CHartographY (SCIAMACHY) and Greenhouse Gas Observing Satellite (GOSAT) for the full tropospheric column measurements (Lacour et al. 2012; Schneider and Hase 2011; Frankenberg et al. 2013). These existing satellites provide global observations of the distribution of water isotopes, providing a unique view on the processes that control the global water cycle. Despite recent advancements, passive remote sensing of HDO in the troposphere using reflected or emitted radiances is typically an ill-posed problem (Galewsky et al. 2016) that limits the accuracy in which vertical distributions can be retrieved in non-trivial heterogeneous scenes (Stevens et al. 2017). Measurements of water isotopes using the measurement approaches presented here are mostly out of the scope of this paper, however, future prospects exist for microwave and LMO.

Hyperspectral passive KID sensors presented here would also extend the capabilities of current infrared sensors for column HDO measurements to cloudy areas with potentially higher spectral resolution, thereby enabling sharper weighting functions in the mid-upper troposphere. LMO also has the potential for retrieving HDO profiles, but similar to the other retrieved products, suffers from poor spatial resolution near the surface. Although column HDO measurements using integrated path DIAL have been demonstrated to date, HDO profile measurements are largely out of scope for active remote sensing such as DIAL and DAR due to the very low atmospheric concentrations. For a HDO DIAL or DAR with similar precision as for H_2_O, for example, one would require a HDO line with an absorption cross section larger by a factor n_H2O/n_HDO than the usual H_2_O DIAL/DAR cross section, where n_x is the molecule number density of isotope x. Due to longer integration paths across the atmosphere, passive remote sensing is in a better position, albeit at the cost of coarse spatial resolution, which may jeopardize the overall benefit as most of the abovementioned hydrological cycle processes are small scale and reside in the mid-to-lower troposphere.

Despite the crucial role of water vapor, and particularly in the lower troposphere, in virtually every climate process (Stevens and Bony 2013), it is important to note that no satellite missions are currently planned which could overcome the limitations of the current deployed space-based sensor suite. The new and emerging technologies and analysis tools presented here have the potential to bridge this observational gap and provide high resolution and accurate water vapor profiles from the surface through the stratosphere. Future climate and dynamics focused Earth observing systems requiring high resolution water vapor profiles will require complementary measurements from DIAL sensors optimized for lower tropospheric measurements as well as from passive sensors whose weighting functions are optimized from the free troposphere up through the stratosphere. The addition of active DAR measurements for in cloud water vapor profiles would complete the portfolio, providing retrievals of water vapor in both clear and cloudy conditions.

In addition to satellite sensors, there is also a need for highly accurate ground-based and airborne remote and in situ measurements of tropospheric water vapor profiles for satellite validation, detailed process studies not achievable using space-borne sensors, and for use as transfer standard to combine satellite datasets. In parallel, the ground-based and airborne lidars and aircraft in situ measurements are vital for looking at detailed micro-/macro-physical processes associated with water vapor and its interaction with clouds. In the near term, airborne process studies such as the European NARVAL and EUREC^4^A campaigns (Kiemle et al. 2017; Bony et al. 2017), will pave the way for next-generation space-based water vapor measurements required to overcome existing measurement deficiencies.
